# Discovery of PF-06928215 as a high affinity inhibitor of cGAS enabled by a novel fluorescence polarization assay

**DOI:** 10.1371/journal.pone.0184843

**Published:** 2017-09-21

**Authors:** Justin Hall, Amy Brault, Fabien Vincent, Shawn Weng, Hong Wang, Darren Dumlao, Ann Aulabaugh, Dikran Aivazian, Dana Castro, Ming Chen, Jeffrey Culp, Ken Dower, Joseph Gardner, Steven Hawrylik, Douglas Golenbock, David Hepworth, Mark Horn, Lyn Jones, Peter Jones, Eicke Latz, Jing Li, Lih-Ling Lin, Wen Lin, David Lin, Frank Lovering, Nootaree Niljanskul, Ryan Nistler, Betsy Pierce, Olga Plotnikova, Daniel Schmitt, Suman Shanker, James Smith, William Snyder, Timothy Subashi, John Trujillo, Edyta Tyminski, Guoxing Wang, Jimson Wong, Bruce Lefker, Leslie Dakin, Karen Leach

**Affiliations:** 1 Medicine Design, Pfizer, Groton, Connecticut, United States of America; 2 Pfizer Centers for Therapeutic Innovation (CTI), Boston, Massachusetts, United States of America; 3 Pfizer Centers for Therapeutic Innovation (CTI), San Diego, California, United States of America; 4 Inflammation and Immunology, Pfizer, Cambridge, Massachusetts, United States of America; 5 External Research Solutions, Pfizer, Groton, Connecticut, United States of America; 6 University of Massachusetts Medical School, Worcester, Massachusetts, United States of America; 7 Medicine Design, Pfizer, Cambridge, Massachusetts, United States of America; 8 Institute of Innate Immunity, University Hospitals Bonn, Bonn, Germany; Toho Daigaku, JAPAN

## Abstract

Cyclic GMP-AMP synthase (cGAS) initiates the innate immune system in response to cytosolic dsDNA. After binding and activation from dsDNA, cGAS uses ATP and GTP to synthesize 2′, 3′ -cGAMP (cGAMP), a cyclic dinucleotide second messenger with mixed 2′-5′ and 3′-5′ phosphodiester bonds. Inappropriate stimulation of cGAS has been implicated in autoimmune disease such as systemic lupus erythematosus, thus inhibition of cGAS may be of therapeutic benefit in some diseases; however, the size and polarity of the cGAS active site makes it a challenging target for the development of conventional substrate-competitive inhibitors. We report here the development of a high affinity (K_D_ = 200 nM) inhibitor from a low affinity fragment hit with supporting biochemical and structural data showing these molecules bind to the cGAS active site. We also report a new high throughput cGAS fluorescence polarization (FP)-based assay to enable the rapid identification and optimization of cGAS inhibitors. This FP assay uses Cy5-labelled cGAMP in combination with a novel high affinity monoclonal antibody that specifically recognizes cGAMP with no cross reactivity to cAMP, cGMP, ATP, or GTP. Given its role in the innate immune response, cGAS is a promising therapeutic target for autoinflammatory disease. Our results demonstrate its druggability, provide a high affinity tool compound, and establish a high throughput assay for the identification of next generation cGAS inhibitors.

## Introduction

The presence of nucleic acids in the cytosol is a danger signal to mammalian cells. This signal initiates activation of innate immunity pathways resulting in the production of interferons and cytokines that comprise the host defense [1–3]. Viral and bacterial infections are well-known sources of foreign RNA and DNA, but self-nucleic acids that have escaped into the cytosol also trigger immune responses, contributing to Type I interferonopathies such as Aicardi-Goutieres syndrome, and systemic lupus erythematosus (SLE) [4–6].

Cyclic GMP-AMP synthase (cGAS) is the most recently identified member of the family of cytosolic DNA sensors. Cytosolic cGAS binds dsDNA and in the presence of ATP and GTP catalyzes the production of the recently characterized second messenger 2′, 3′- cyclic AMP-GMP (cGAMP) which then binds to Stimulator of Interferon Genes (STING). The cGAS /STING dyad appears to be ancient, with homologs co-evolving from unicellular organisms over 500 million years distant from humans; the strength of the conservation pressure on the cGAS/STING dyad may be illustrative of their importance to cellular defense and immunity [7, 8]. In humans, the binding of the cGAS product to STING causes a conformational change resulting in recruitment of TBK1, and interferon-inducible gene activation and interferon production via IRF3 phosphorylation and nuclear translocation [9–12]. A number of other cytosolic DNA sensors exist, including Absent in Melanoma 2 (AIM2), DNA-dependent activator of IRFs (DAI) and IFN-γ-inducible protein 16 (IFI16) but accumulating evidence suggests cGAS is the primary sensor in innate immune activation [13–17].

Activation of cGAS is important in host defense against pathogens, but uncontrolled activation of the cGAS pathway has been implicated in autoinflammatory disease. For example, gain-of-function mutations in STING result in the autoinflammatory disease SAVI (STING-associated vasculopathy with onset in infancy), characterized by interferonopathy resulting in skin lesions, interstitial lung disease, and systemic inflammation [18]. Self-DNA normally is absent from the cytosol due to the primary mammalian exonuclease TREX1. TREX1 is one of seven human genes whose mutation cause Aicardi-Goutieres syndrome (AGS), a severe inflammatory disease, and a small percentage of SLE patients have TREX1 mutations [19–21]. TREX1 knockout mice have elevated levels of dsDNA, elevated levels of cGAMP, and display multiorgan inflammation (especially myocarditis) leading to morbidity [22, 23]. The double TREX1/cGAS knockout rescues the TREX1 phenotype, demonstrating a key role for cGAS stimulation in autoinflammation [24, 25]. Elevated levels of cGAMP have been reported recently in a subset of SLE patients with a more severe disease phenotype (as shown by higher SLEDAI scores) compared to SLE patients in whom no cGAMP was detected [26]. Taken together, these results support dysregulation of the cGAS/STING signaling axis in several autoimmune diseases.

The evidence linking activation of the cGAS pathway to autoimmune disease suggests that cGAS inhibitors may have therapeutic efficacy. Few inhibitors have been identified, hampered in part by the lack of sensitive, high throughput screening assays. Although DNA-binding compounds may indirectly inhibit cGAS activity, to our knowledge no inhibitor shown to bind directly to the cGAS active site has been reported. To discover cGAS active site inhibitors we used NMR screening of a fragment library and identified a compound that binds competitively with cGAMP. Structure-based drug design and chemical optimization of the original fragment hit resulted in a high affinity lead that binds in the nucleotide binding site and inhibits cGAS activity. To address the need for screening assays we produced a novel cGAMP mAb and developed a high throughput FP assay which can detect cGAMP produced by cGAS concentrations at levels as low as 10 nM. Taken together our results demonstrate the druggability of cGAS, provide a high affinity active site inhibitor and establish a sensitive screening assay with broad utility for cGAS drug discovery.

## Materials and methods

Additional details of the Methods are provided in the Supporting Information. PF-06928215 was synthesized in house according to procedures in the Methods section; PF-06928215 is commercially available via Sigma-Aldrich (catalog number PZ038). The cGAMP monoclonal antibody 80–2 (**PF-07043030)** will be commercially available from Sigma.

ISD DNA (catalog number tlrl-isdn), ATP, GTP, cAMP, cGMP, cGAMP were purchased from Invivogen, Donkey anti mouse-IgG-HRP from Jackson ImmunoResearch, BD OptEIA Substrate Reagent Set Cy5 mono NHS ester from GE Healthcare Life Sciences, N,N-diisopropylethylamine from Applied Biosystems, N-methyl-2-pyrrolidone from Applied Biosystems, trifluoroacetic acid (TFA) from Applied Biosystems and acetonitrile (HPLC grade) from Fisher Scientific. PPD was purchased from Cambridge Research Biomolecules, BSA from Thermo Fisher Scientific, Streptavidin from Thermo Fisher Scientific, sodium bicarbonate from Sigma-Aldrich, dimethyl sulfoxide from Sigma-Aldrich and Slide-A-lyzer cassettes from Thermo Fisher Scientific.

### cGAS activity mass spectrometry assay

cGAS activity was measured in buffer (10 mM Hepes, 140 mM NaCl, 0.01% Tween-20, 5 mM MgCl_2,_ pH 7.5) containing 1 mM ATP, 0.3 mM GTP, 100 nM ISD DNA (45 bp dsDNA), and 100 nM cGAS in the presence or absence of test compounds. For K_m_ determinations, the conditions were modified to 1 mM ATP and varying amounts of GTP or 0.3 mM GTP and varying amounts of ATP. The reaction was incubated for 30 minutes at 37°C and stopped with the addition of 50 mM EDTA. 2’3’-cGAMP was measured by mass spectrometry analysis. An earlier version of the assay utilized the same buffer and nucleotide conditions but was conducted in the presence of 1 nM cGAS, 1 nM ISD DNA and carried out for 6 h at 37°C before being processed as described above.

### Mass spectrometry analysis

50 μl of H_2_O was added to each 25 μl cGAS reaction (384 well format). The samples were mixed by briefly vortexing followed by a 10 min, 3000 g centrifugation at room temperature. 2,3 cGAMP was monitored using 1290 Agilent UPLC in conjunction a Sciex 5500 triple-quadrupole mass spectrometer in MRM mode. 10 μl of sample was injected on to a 2x30 mm hypercarb column (Thermo Scientific) kept at 60°C with a flow rate of 0.8 ml/min and a loop sweep time of 20 s. Mobile phase A consisted of 10 mM Ammonium Acetate, pH 10, and Mobile phase B consisted of 90:10 (Acetonitrile:Acetone 1:1): 10 mM Ammonium Acetate pH 10 + 0.1% formic acid. The LC gradient used to separate 2,3 cGAMP is as follows: initial conditions: 85% A, 15% B; Time 0.1: 85% A, 15% B; Time 0.2: 30% A, 70% B; Time 0.3: 30% A, 70% B; Time 0.35: 85% A, 15% B; Time 0.4: 85% A, 15% B. The LC cycle time (time/sample run) equaled 30 s. The LC eluent was diverted to waste for the initial 12 s before monitoring 2,3 cGAMP via MS-MRM. 2,3 cGAMP was monitored using 3 MRM transitions: 675.1–506.1 m/z (primary), 675.1–524.3 m/z (secondary), 675.1–136.2 m/z (secondary) with the optimized parameters: MRM parameters: dp 99 ep 10 ce 36 cxp 10 for the 506.1 transition and dp 99 ep 10 ce 64 cxp 10 for the 524.3 and 136.2 m/z transitions. The substrates ATP and GTP were monitored as loading controls with the following MRM transitions: 508.1–136.2 (ATP) and 524.0–152.0 (GTP), respectively. ATP and GTP had the following optimized parameters: dp 99 ep 10 ce 64 cxp 10. The mass spectrometer was set with the following parameters: time/MRM: 50 msec, cycle time: 0.2750 s, delay time: 0, resolution: unit/unit, pause between mass range: 5.007 msec, curtain gas: 35, collision gas: 10, ion spray voltage: 5500 temp: 600°C, ion source gas 1: 60, ion source gas 2: 60. The data was processed using Sciex's Multiquant 3.0.

### cGAMP mAb

All animal work, care, and housing was provided by ProSci Incorporated Mice were provided daily veterinary supervision and care; bleedings were performed without analgesics, Fatal-Plus was used for euthanasia. Mice were 8 week old at immunization, 5 A/J mice and 5 SJL/J mice (The Jackson Laboratory) were injected with a 2:1 mixture of the cGAMP compounds linked at the adenine NH_2_, PPD-cGAMP and Streptavidin-cGAMP, and Complete Freud’s Adjuvant, subcutaneously, for the first immunization only. The mice were subsequently boosted three more times as above, except with Incomplete Freund’s Adjuvant, spaced every three weeks. Mouse sera titers were determined by screening for binding to cGAMP-BSA derivatives coated on a plate in a DELFIA immunoassay (Perkin Elmer). Once a high titer was detected the mouse selected for fusion was given an intraperitoneal final boost with a 2:1 mixture of the two cGAMP analogues above, no adjuvant, four days prior to the fusion. Hybridomas were derived by a fusion of mouse splenocytes from a high titer mouse with the mouse myeloma cell NS-1 (ATCC). Mouse spleen cells were isolated, the red blood cells were lysed in Red Blood Cell lysing buffer (Sigma), and the splenocytes were depleted of B cells expressing surface IgM by using magnetically labeled anti-mouse IgM micro beads (MAC Miltenyl Biotec). The cells were washed twice in IMDM media (Gibco) and pelleted with the NS-1 cells at a 4:1 ratio. A solution of 50% PEG (Sigma) was added to the pellet with gentle shaking. The cells were plated in 96 well plates in IMDM media containing HAT for one week before the hybridomas were screened.

### cGAMP antibody production and purification

Hybridoma supernatants were screened for binding to BSA-cGAMP derivatives by DELFIA immunoassay. Specificity for cGAMP over cAMP and cGMP was determined by DELFIA using biotin-cAMP (Biotium) and biotin-cGMP (Biotium) on streptavidin assay plates (Pierce). Hybridomas specific for cGAMP were cloned one time by limiting dilution and expanded for antibody production. The hybridomas were grown in IMDM culture media containing low IgG fetal bovine serum (Hyclone). Antibodies were purified from conditioned hybridoma media by protein A/G chromatography followed by size-exclusion chromatography. Samples were concentrated to approximately 5mg/mL and dialyzed against 20 mM HEPES, 150 mM NaCl, pH 7. Final samples were analyzed for percent monomer, percent purity, concentration and stability after one freeze-thaw.

### cGAMP ELISA assays

Costar high binding plates were coated with BSA-cGAMP (50 ng/well) in PBS (without calcium or magnesium) and incubated overnight at 4°C. Plates were washed with buffer (TBS containing 0.05% Tween-20) and blocked for 2 hr at room temperature with Assay buffer (TBS containing 0.05% Tween 20 and 1% BSA) followed by washing. cGAMP mAb (diluted in assay buffer) was added to plates for 1 hr, followed by washing and incubation with donkey anti-mouse IgG-HRP for an additional hr. After washing, plates were developed with substrate, the reaction was stopped with 2 N H_2_SO_4_ and OD was read at 450 nm. The competition ELISA assays followed the same procedure, with the exception that 40 ng/ml anti-cGAMP mAb was preincubated with various concentrations of cGAMP, ATP or GTP for 1 hr prior to addition to the BSA-cGAMP assay plates. After incubation for 1 hr, plates were washed, incubated with secondary antibody, and developed as described above.

### Cy5-cGAMP fluorescence polarization assay

cGAS activity was measured in buffer (10 mM Hepes, 140 mM NaCl, 0.01% Tween-20, 5 mM MgCl_2,_ pH 7.5) containing 1 mM ATP, 0.3 mM GTP, 100 nM ISD DNA (45 bp dsDNA), and 100 nM cGAS. For inhibition assays, compounds were included in the reaction, and after incubation for 1 h at room temperature, reactions were stopped with 50 mM EDTA followed by addition of Cy5-labeled cGAMP (2 nM) and 16 nM cGAMP antibody. After incubation for 1 h at room temperature, plates were read on an Envision plate reader (excitation: 620 nm; emission: 688 nm). Determination of the Ki of cGAMP for mAb 80–2 was conducted with the following equation, specifically derived for fluorescence polarization assays [27].

$$\text{Ki}={\left[\text{I}\right]}_{50}/({\left[\text{L}\right]}_{50}/\text{Kd}\;+\;{\left[\text{P}\right]}_{0}/\text{Kd}\;+\;1)$$

## Results and discussion

### Characterization of cGAS enzymatic activity

**Fig 1:**
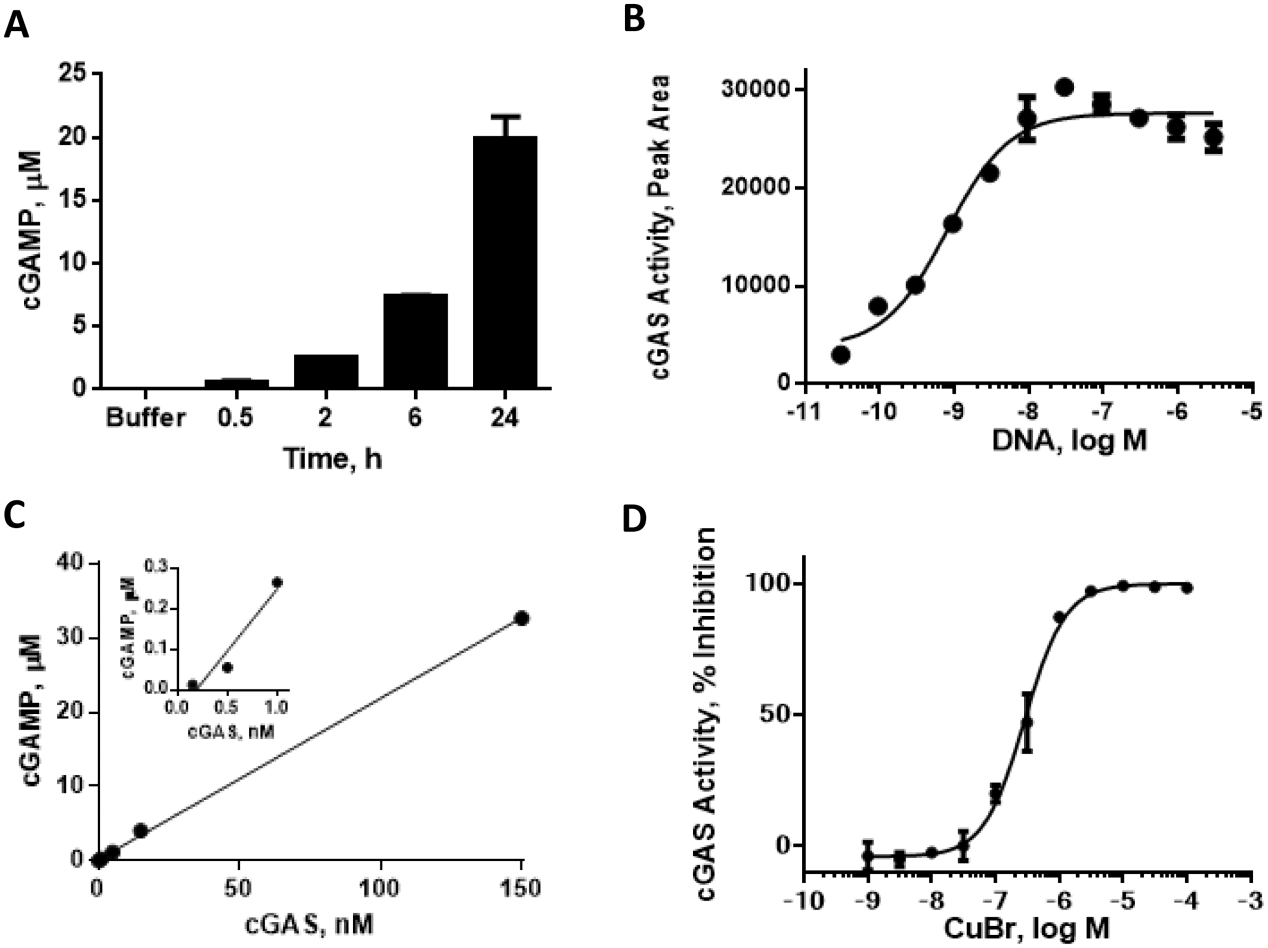
Characterization of cGAS enzyme activity. Measurement of cGAMP production was conducted by LC-MS as described in Methods. (A) Time course of cGAS (15 nM) activity; (B) titration of dsDNA activation of cGAS (1nM) activity; (C) cGAS enzyme titration; (D) inhibition of cGAS (1 nM) activity by CuBr.

In the presence of ATP, GTP and dsDNA, cGAS catalyzes the production of the novel second messenger cGAMP. cGAMP levels can be measured directly via mass spectrometry, and we initially used this medium-throughput format to establish enzyme assay conditions after optimizing incubation time, and the concentration of DNA, substrates and enzyme (Fig 1A–1C). Briefly, cGAMP was separated from enzymatic assay reaction using liquid chromatography and its levels measured using mass spectrometry. Human cGAS (aa 2–522) produced and purified from Sf9 cells displayed concentration-dependent increases in cGAMP production. cGAS activity was typically measured using 100 nM enzyme though concentrations as low as 1 nM resulted in detectable levels of cGAMP. The assay was linear for over 6 hr at 37°C and a 30 min reaction time was used routinely for testing compounds at the 100 nM enzyme concentration. As expected, cGAS activity was dependent on dsDNA; with 1 nM cGAS, an EC_50_ value of 1 nM was obtained for dsDNA suggesting a high affinity interaction. Higher cGAS concentrations required stoichiometrically higher DNA concentrations for maximal activation (data not shown). We generally used the 100 nM cGAS assay as we found the 1 nM version was prone to a high false positive rate. For example, the 1 nM cGAS assay conducted with 1 nM dsDNA was very sensitive to inhibition by metals (including copper and palladium) commonly used as catalysts in chemical synthesis while zinc addition lead to increased enzymatic activity (Fig 1D and S1 Fig). This high false positive rate was significantly reduced using 100 nM cGAS with 100 nM dsDNA though hit re-purification or re-synthesis was essential for validation.

cGAS requires both ATP and GTP for catalysis but the K_m_ values for the nucleotides have not been reported to date. Apparent K_m_ values were determined by varying the concentration of one nucleotide in the assay while keeping the other nucleotide fixed (S2 Fig). The fixed concentrations of ATP and GTP roughly approximated physiological levels at 1 mM and 300 μM, respectively. Enzymatic activity increased sharply with increasing nucleotide concentrations, while concentrations greater than 1 mM resulted in decreased cGAMP production, consistent with substrate inhibition. Under these conditions the apparent K_m_ values were similar, 30 μM for GTP and 35 μM for ATP. We have carried out a deeper kinetic study of the cGAS mechanism using multiple assay formats which we report in detail elsewhere (manuscript submitted), and our general results are in agreement with the apparent K_m_ and substrate inhibition effects we describe here.

### Development of a high throughput cGAS fluorescence polarization assay

The mass spectrometry assay has the advantage of directly measuring the cGAMP product, but it is generally a slower and more resource-intensive medium-throughput assay. Therefore, to enable high throughput screening capabilities of larger compound sets we developed a fluorescence-based assay that could be utilized to screen for cGAS inhibitors. The FP assay requires an antibody specific for cGAMP as well as a fluorescently labelled cGAMP analog. As such, an ethylenediamine-cGAMP analog (Compound **11**) was synthesized as a versatile intermediate that enabled antibody generation (S3 Fig).

A number of different cGAMP conjugates were synthesized for mouse immunizations and antibody screening (S4 Fig and Supporting Information). Mice were immunized with a mixture of cGAMP-PPD and cGAMP-SA, and serum was tested in a DELFIA immunoassay for reactivity against a further analog, cGAMP-BSA. This screening and immunization strategy allowed for the selection of mAbs that specifically recognized cGAMP while excluding mAbs against either the PPD and biotin-streptavidin conjugates, or the PEG linkers.

Hybridomas were derived from mice with positive titers, and supernatants were screened against cGAMP-BSA. Selectivity for cGAMP was an important requirement for the final mAb and therefore positive supernatants were counterscreened against biotin-cAMP and biotin-cGMP. Hybridomas specific for cGAMP were cloned by limiting dilution, and mAb 80–2 was selected for further characterization and FP assay development. In an ELISA assay against BSA-cGAMP, mAb 80–2 displayed an EC_50_ value of 40 pM and was specific for cGAMP as demonstrated in competition experiments with ATP, GTP, cAMP and cGAMP (S5 Fig and data not shown).

**Fig 2:**
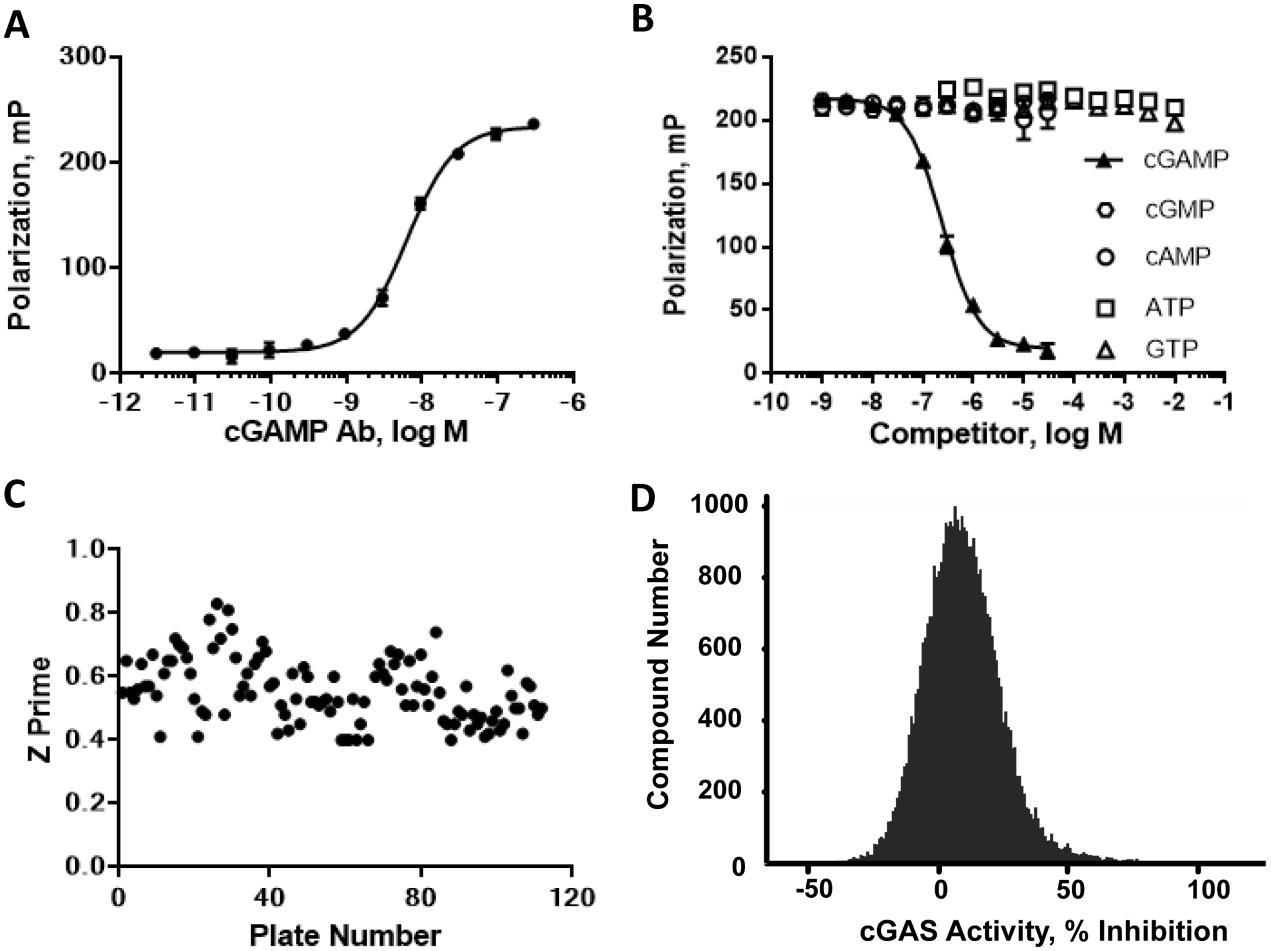
Characterization of cGAMP FP assay. (A) mAb titration with Cy5-cGAMP (2 nM); (B) competition of Cy5-cGAMP (2 nM) binding to mAb 80–2 with: cGAMP, cAMP, cGMP, ATP or GTP; (C) Z’ results of FP assay in subset screen; (D) Distribution of compound activity from subset screen.

For the FP competition assay, the ethylenediamine-cGAMP (**11**) was labelled through the primary amine with Cy5, a long wavelength fluorescent dye. Titration of mAb 80–2 with 2 nM Cy5-cGAMP demonstrated an EC_50_ of ~8 nM; 2 nM Cy5-cGAMP and 16 nM mAb 80–2 were chosen as the optimal concentrations for the cGAS assay (Fig 2A). Cy5-cGAMP binding to mAb 80–2 was specific, and was competed by cGAMP, but not by ATP, GTP, cAMP or cGMP (Fig 2B). This demonstration of specificity was critical to establish since ATP and GTP are high concentration substrates in the cGAS enzyme assay and could potentially interfere with the FP signal. The fluorescence polarization data does suggest that the significantly higher affinity observed in the ELISA assay (40 pM) is likely an overestimation of antibody affinity. Additionally, as cGAMP displayed a K_i_ of 76 nM (Fig 2B) for mAb 80–2 in competition mode, we cannot rule out that this antibody may recognize the linker region in addition to cGAMP itself. Nonetheless, the assay was established and optimized in 384-well format, with a Z′ of >0.5 and a robust signal/background of >80 mP (Fig 2C). A screen of forty thousand compounds at a final concentration of 100 μM displayed a normal distribution for the frequency of compound inhibition (Fig 2D). Additionally, a test set of approximately 40 compounds including initial fragment hits and related compounds that showed activity in the mass spectrometry assay was used to validate the FP assay; there was less than 3-fold variation in the IC_50_ values obtained between the mass spectrometry method and the FP assay (data not shown). The FP assay has several advantages compared to the more resource intensive mass spectrometry approach and proved to be a high throughput, rapid and robust assay for detecting cGAS inhibitors in an enzyme assay. Recently, a riboswitch cGAMP biosensor has been reported that was proposed to be suitable for high throughput screening (HTS) and used to quantify micromolar cellular cGAMP levels in cells overexpressing cGAS [28]. We attempted to utilize the FP assay format to detect cGAMP levels in dsDNA-stimulated THP-1 cells, but were not able to reproducibly measure cellular cGAMP using this approach (data not shown). Overall, these results demonstrate the utility of mAb 80–2 and the FP assay for HTS purposes while they also highlight their limitations in terms of measurement of endogenous cGAMP levels in primary human cells.

### Discovery and optimization of an active site inhibitor

The catalytic mechanism of cGAMP formation by cGAS has been reported previously. Briefly, ATP and GTP are simultaneously bound, dsDNA activates cGAS and selectively orients substrates through Thr_211_ and Arg_376_ (*Homo sapiens* numbering) at the guanine base [29] and the adenine ribose through Asp_227_ [30] while priming catalysis at the ATP α-phosphate and the 2´-OH of the guanine ribose to form the linear GTP-2´-3´-AMP intermediate. The intermediate must flip over to allow a similar catalytic event to occur between the guanine 5´-α-phosphate and the 3´-OH of the adenine ribose, making the final cyclic dinucleotide 2´,3´-cGAMP [31]. cGAS can accommodate the rearrangements of the intermediates because the active site is both large and polar, which can present challenges to the identification and optimization of high affinity small molecule ligands and inhibitors. Consistent with this observation, computational scoring of the cGAS active site using several different methods [32–34] suggests this pocket is marginally druggable (data not shown).

**Fig 3:**
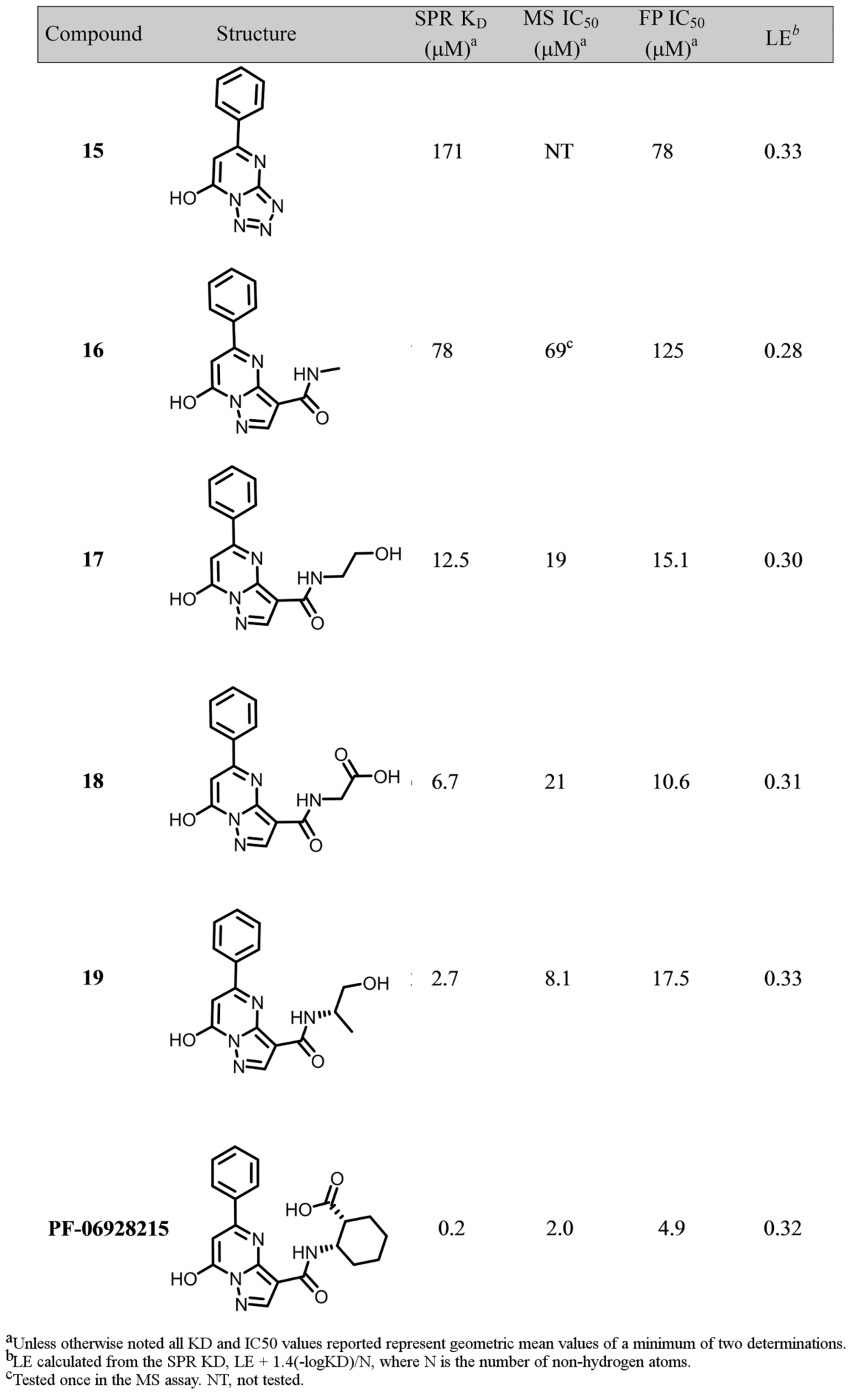
Binding affinities and *in vitro* activities of cGAS inhibitors.

**Fig 4:**
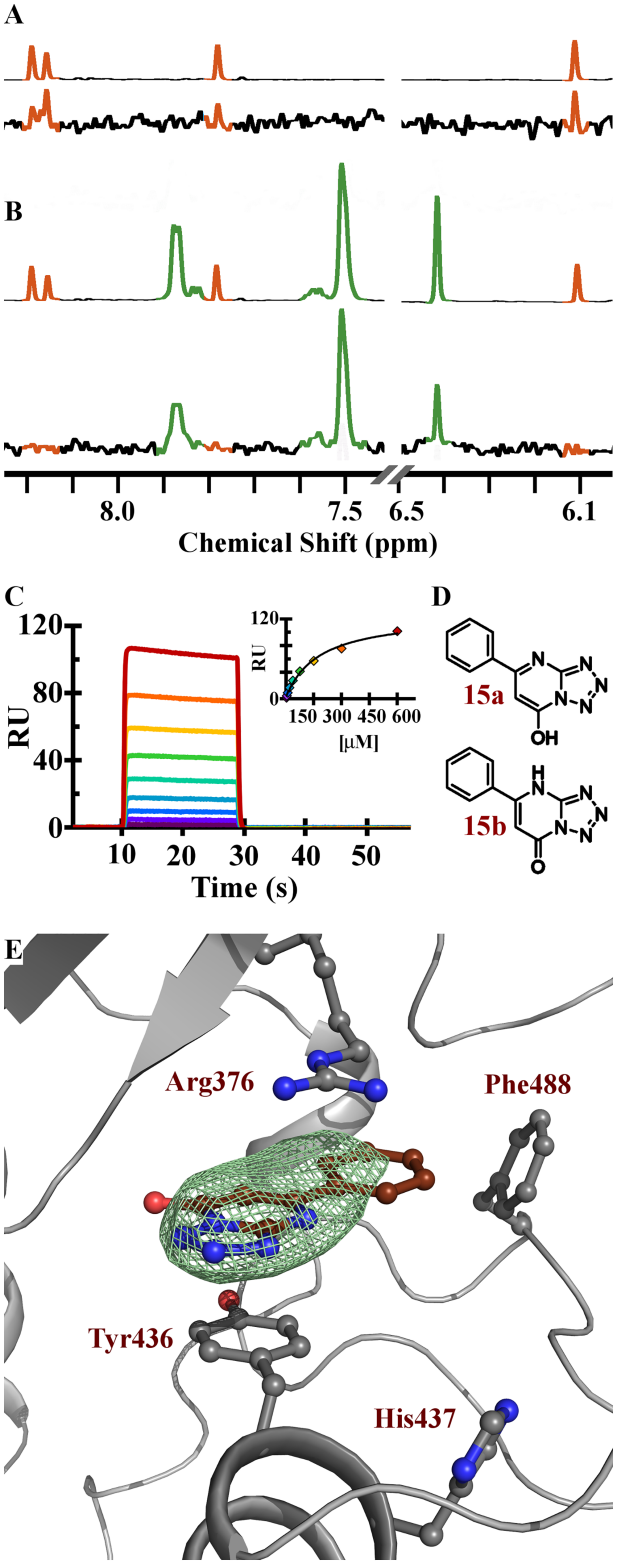
Characterization of compound 15 binding to cGAS. (A) 1D ^1^H spectra of 2´,3´-cGAMP (top) and ^1^H STD of 2´,3´-cGAMP interacting with cGAS (bottom). (B) 1D ^1^H spectra of 2´,3´-cGAMP (orange) and compound 15 (green) (top) and ^1^H STD of a mixture of 2´,3´-cGAMP and compound **15** showing compound **15** has out competed 2´,3´-cGAMP for interacting with cGAS (bottom). (C) SPR sensorgram of compound **15** with binding fit inset. (D) Compound **15** in either its hydroxyl (15a) or keto (15b) tautomeric forms. (E) cGAS active site showing residues that interact with compound **15**; Fo-Fc electron density omit map (green) for compound **15** (brown) is contoured at 3 Sigma and shows all density within 4 Å of compound **15**.

Nonetheless, despite its relatively open and polar character, the cGAS active site is calculated to be the most druggable pocket on the protein. In an effort to identify cGAS ligands we undertook a saturation transfer difference (STD) ^1^H NMR screen of the Pfizer fragment library against a previously crystalized cGAS construct beginning at residue 161 (cGAS_161_) (*H*. *sapiens* numbering); details of the library and general strategy have been described previously [35]. To identify the active site ligands we subsequently rescreened the initial binders in the presence of 2´,3´-cGAMP and prioritized those that displaced the enzymatic product. The resulting fragments were then tested for binding affinity to cGAS by SPR in the absence of activating dsDNA. Finally, we attempted to obtain structures of the tightest binders to cGAS_161_. Compound **15** was identified via the NMR screen, and SPR demonstrated that it bound directly to cGAS, although with low affinity (K_D_ = 171 μM) (Figs 3, 4A and 4B). In the FP assay, compound **15** was detected as a weak inhibitor, with an IC_50_ of 78 μM against cGAS enzymatic activity.

The X-ray structure of compound **15** confirmed that it binds in the active site of cGAS (Fig 4C and S1 Table for crystallographic statistics). The binding site of **15** is formed between the plane of the phenyl ring of Tyr_436_ and the guanidyl group of Arg_376_. Based on published X-ray structures of cGAS, compound **15** is occupying a similar region of the adenine base binding site of ATP [30] or 2´,3´-cGAMP (see PDB 4K9B [31] and 4O67 [36]).

The tetrazole-containing biaryl ring of compound **15** accounts for the majority of the interaction with Tyr_**436**_ and Arg_376_, yet modification or deletion of the phenyl ring of compound **15** resulted in complete ablation of binding suggesting an important role for the phenyl moiety in compound affinity (data not shown). The requirement of the phenyl of compound **15** may be the result of an edge-face interaction of the phenyl with the side-chain of Phe_488_ (*ca* 3.35 Ǻ). In fact, the electron density of **15** suggests the phenyl is coplanar with the tetrazolopyrimidine aromatic core. In order to maintain a reasonable torsional strain of this co-planarity the phenolic tautomer of **15** (OH) is likely the preferred tautomer in the bound state (Fig 4D, 15a).

**Fig 5:**
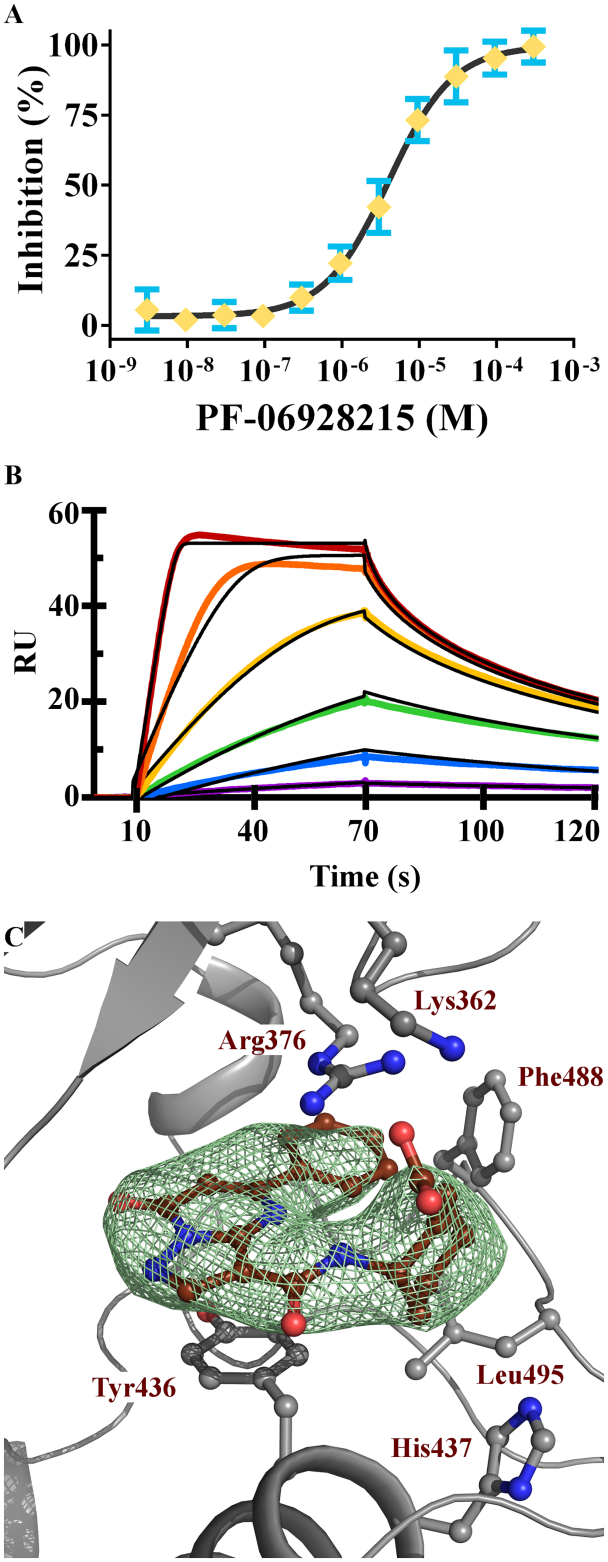
PF-06928215 is a high affinity cGAS inhibitor. (A) Concentration-dependent inhibition in cGAMP FP assay. (B) SPR sensorgram of **PF-06928215** binding to cGAS, 2-fold variations in the concentration of **PF-06928215** are shown in rainbow registry with kinetic association and dissociation fits in black. (C) cGAS active site showing residues that interact with **PF-06928215**, Fo-Fc electron density omit map (green) for **PF-06928215** (brown) is contoured at 3 Sigma and shows all density within 4 Å of **PF-06928215**. Figure was generated using Pymol.

Compound **15** was pursued further due to its high ligand efficiency and competition with 2´,3´-cGAMP. However, it was discovered upon attempted resynthesis that this compound is not chemically stable and readily isomerizes via an open-ring azidopyrimidine (S5 Fig) [37]. From a chemical optimization perspective this was not attractive and thus several chemically stable [6,5] ring systems were synthesized in an attempt to find a core that would recapitulate the active pharmacophore and not isomerize. In this process we found the original tetrazolopyrimidine core of **15** could be substituted for a pyrazolopyrimidine (**16**) which maintained comparable affinity (K_D_ = 78 μM, and inhibition of cGAS functional activity (IC_50_ = 69–125 μM) as the original fragment hit (Fig 3). The X-ray structure of the pyrazolopyrimidine **16** was solved and it bound in a similar manner to the original fragment hit **15**. Notably, the regioisomeric analog **16** was also prepared and it did not demonstrate binding affinity to cGAS (data not shown). The structure of compound **16** shows the amide substituent on the pyrazole afforded an attractive vector to explore other regions of the active site. We found that amides capped with polar functionalities were favored and demonstrated significant increases in both the SPR affinity and the functional activity of these inhibitors. Compounds **17** and **18** inhibited cGAS activity with IC_50_ values below 20 μM (Fig 3). Notably, compound **19** and **PF-06928215** have binding affinities of 2.7 and 0.2 μM, respectively, with a corresponding increase in inhibitory potency in the cGAS assay; **PF-06928215** inhibited cGAS activity with an IC_50_ value of 4.9 μM (Fig 5A). As with our other compounds, there is good agreement between binding and inhibition, though activity measurements occur in the presences of competing nucleotides which would cause the IC_50_ to appear weaker than K_D_ measurements conducted without nucleotides. Compared to compound **15**, **PF-06928215** has improved in potency and displays longer off rate kinetics than any other compound in this series (Figs 4C and 5B).

Despite the progress in biochemical potency, **PF-06928215** displayed no activity in cellular cGAS assays measuring dsDNA-induced IFN-beta expression via a luciferase reporter (Panel A in S7 Fig). Compounds **17–19** also had no effect on IFN-β induction. Notably, the TBK1 inhibitor BX-395 was used as a positive control, and inhibited induction with an IC_50_ value of ~ 60 nM. The lack of inhibitory activity of the **PF-06928215** and related compounds was not the result of cytotoxicity, as the compounds had minimal effect on cell viability (Panel B in S7 Fig). The reason(s) for the compounds' inability to inhibit IFN induction is not known but considering the high levels of ATP and GTP, it is likely that further improvements in potency are required for inhibition of cellular cGAS activity. Additionally, to improve this class of biochemical inhibitors improvements to their passive permeability and plasma protein binding profiles are required. Replacement of the carboxylic acid with an isostere that has a higher pKa, thus lowering the polar surface area, and reducing the number hydrogen bond donors are two possible approaches.

Subsequent X-ray structures of **PF-06928215** show the alkyl chain interacting with a small hydrophobic pocket formed between Tyr_436_ and His_437_ while the polar head groups prefer to interact with the polar residues Arg_376_ and Lys_362_ above the biaryl core (Fig 5C). In **PF-06928215** the hydrophobic interactions extend to Leu_495_ and the carboxylate head piece also interacts with a partially ordered Lys_362_. Interestingly, when comparing the interactions of compound **PF-06928215** with reported structures of cGAS, we found that the Lys_362_ interaction of **PF-06928215** closely mimics an interaction with Lys_350_ (*Mus*. *musculus* numbering) in the linear 5´-pG (2´-5´)pA pseudo-intermediate reported by the Patel lab [31] (PDB 4K9A). In 4K9A, Lys_350_ interacts with an oxygen of the 2´-5´ phosphodiester bond, however in none of the cyclic dinucleotide structures is this Lys induced to reach the phosphodiester bond. Thus we speculate that this interaction is particular to how cGAS holds the linear intermediate during the catalytic process. **PF-06928215** might be mimicking this interaction in the active site (Fig 5C**)**.

The newly reported dsDNA sensing enzyme cGAS has emerged as a new player in the innate immunity pathway. Direct genetic links of cGAS to human disease have not been shown, but gene knockouts in animal models have demonstrated the importance of cGAS in controlling immune activation. These results have important implications suggesting a role of cGAS in immune diseases and highlight the need for pharmacological tools to probe cGAS function in cells and animal models. Despite a large and polar active site, our results demonstrate that active site inhibitors can be identified and chemistry optimization resulted in an inhibitor with nM binding affinity and μM functional activity. The development of the high throughput FP assay to measure cGAS activity can be readily utilized for screening large compound sets and should be enabling for the further discovery of potent, cGAS inhibitors with cellular activity which would be useful in further elucidating the role of this enzyme in disease.

## Supporting information

S1 Fig Modulation of cGAS enzyme activity by metals in the MS assay. (A) Inhibition dose response of Pd(OAc)_2_ with cGAS (1 nM); (B) Activation dose response of ZnCl_2_ with cGAS (1 nM) and ISD dsDNA (1nM); NB: lower activation multiples were observed with higher ISD DNA concentrations (data not shown); (C) Results from a panel of metal salts tested in the cGAS (1nM) assay in the presence of 1 nM ISD DNA.
(DOCX)

S2 Fig Nucleotide-dependence of cGAS enzyme activity. (A) Apparent K_m_ determination for GTP in the presence of 1 mM ATP; (B) Apparent K_m_ determination for ATP in the presence of 0.3 mM GTP.
(DOCX)

S3 Fig Preparation of a cGAMP analog containing an ethylenediamine functionality. A protected and activated ethylenediamine adenosine analog was prepared (**5**) and coupled with a protected guanosine analog (**7**) in acetonitrile, followed by oxidation to the phosphate (**8**). Protecting group manipulation, cyclisation and further oxidation provided the protected cGAMP analog (**9**). Stepwise removal of the various protecting groups then provided the desired ethylenediamine-cGAMP analog (**11**). Compound **11** proved to be a highly useful intermediate, whereby the primary alkyl amino group could be selectively reacted with various linker groups, forming a stable amide bond, followed by subsequent conjugation or binding to various proteins for antibody generation or screening.
(DOCX)

S4 Fig cGAMP derivatives for mAb production and screening. (A) An ethylenediamine-cGAMP analog linked through PEG5 to a reactive NHS ester (**13**) for subsequent attachment to PPD (cGAMP-PPD); (B) an ethylenediamine-cGAMP analog linked through PEG6 to a biotin molecule (**14**) for subsequent binding to streptavidin (cGAMP-strepavidin) were synthesized. Mice were immunized with a mixture of these protein conjugates. (C) serum was tested in a DELFIA immunoassay for reactivity against a further analog, ethylenediamine-cGAMP linked through C6 to a reactive NHS ester (**12**) which allowed conjugation to BSA (cGAMP-BSA). (D) an ethylenediamine-cGAMP analog conjugated to Cy5 was synthesized to be used as the fluorescently labelled cGAMP analogue in the FP assay.
(DOCX)

S5 Fig cGAMP mAb 80–2 characterization. (A) titration of mAb 80–2 in cGAMP ELISA; (B) mAb 80–2 was preincubated with cGAMP, ATP or GTP for 1 hr prior to addition to the cGAMP-BSA coated assay plates. Binding was inhibited in a concentration-dependent manner by cGAMP, but neither ATP nor GTP at mM concentrations inhibited the binding of mAb 80–2 to BSA-cGAMP. Data points are average of duplicate determinations; error bars represent standard deviation.
(DOCX)

S6 Fig Compound 15 can readily isomerize via ring opening through an open azidopyrimidine. (DOCX)

S7 Fig Effect of cGAS inhibitors on IFNβ induction. THP-1 Dual cells were pretreated with various concentrations of BX-795 (red triangles), Compound 17 (yellow squres), Compound 18 (purple triangles), Compound 19 (black triangles) or PF-06928215 (blue circles) for 1 hr followed by stimulation with salmon sperm DNA for 12 hrs. Media was collected and analyzed for luciferase signal (A), and cell viability (B) was analyzed with CellTiter Glo, as described in Methods.
(DOCX)

S1 Table Crystallographic data and refinement statistics. (DOCX)
