# Supplementary material for: Discovery of PF-06928215 as a high affinity inhibitor of cGAS enabled by a novel fluorescence polarization assay
Source: PLoS One. 2017 Sep 21;12(9):e0184843. doi: 10.1371/journal.pone.0184843 (PMC5608272; doi:10.1371/journal.pone.0184843)
Supplement: S1 Fig — (A) Inhibition dose response of Pd(OAc)2 with cGAS (1 nM); (B) Activation dose response of ZnCl2 with cGAS (1 nM) and ISD dsDNA (1nM); NB: lower activation multiples were observed with higher ISD DNA concentrations (data not shown); (C) Results from a panel of metal salts tested in the cGAS (1nM) assay in the presence of 1 nM ISD DNA. (DOCX) [file pone.0184843.s001.docx]

**SUPPORTING INFORMATION**

**Discovery of PF-06928215 as a high affinity inhibitor of cGAS enabled by a novel fluorescence polarization assay**

Justin D. Hall^1*^, Amy Brault^1^, Fabien Vincent^1^, Shawn Weng^2^, Hong Wang^1^, Darren Dumlao^1^, Ann E. Aulabaugh^1^, Dikran Aivazian^3^, Dana Castro^3^, Ming Chen^1^, Jeffrey S. Culp^1^, Ken Dower^4^, Joseph P. Gardner^5^ , Steven J. Hawrylik^1^, Douglas Golenbock^6^ David Hepworth^7^, Mark Horn^3^, Lyn H. Jones^7^ , Peter Jones^7^, Eicke Latz^6,8^, Jing Li^7^, Lih-Ling Lin^4^, Wen Lin^1^, David W. Lin^1^, Frank Lovering^7^, Nootaree Niljanskul^1^, Ryan Nistler^2^, Betsy Pierce^1^, Olga Plotnikova^1^, Daniel C. Schmitt^1^, Suman Shanker^1^, James F. Smith^1^, William B. Snyder^3^, Timothy Subashi^1^, John I. Trujillo^1^, Edyta Tyminski^2^, Guoxing Wang^2^ Jimson Wong^1^, Bruce A. Lefker^7^, Leslie A. Dakin^7^, Karen L. Leach^2^

^1^Medicine Design, Pfizer, Eastern Point Rd, Groton, CT 06340

^2^Pfizer Centers for Therapeutic Innovation (CTI), 3 Blackfan Circle, Boston, MA 02115

^3^Pfizer Centers for Therapeutic Innovation (CTI), 10770 Science Center Dr, San Diego, CA 92121

^4^Inflammation and Immunology, Pfizer, 610 Main St, Cambridge, MA 02115

^5^External Research Solutions, Pfizer, Eastern Point Rd, Groton, CT 06340

^6^University of Massachusetts Medical School, Worcester, MA Worcester, MA 01605

^7^Medicine Design, Pfizer, 610 Main St, Cambridge, MA 02139

^8^Institute of Innate Immunity, University Hospitals Bonn, Bonn, Germany

^*^Corresponding author

^.^

**METHODS**

**cGAS production: Vector Construction.** Amino acids 2-522 of human MB21D1 (Uniprot ID: Q8N884; codon optimized for *Spodoptera frugiperda*) was synthesized at GeneArt AG (LifeTechnologies/Thermo-Fisher) and sub-cloned to the baculovirus transfer vector pFastBac1 via BamHI and EcoRI restriction sites. A 6X HIS tag and TEV protease cleavage site (MGSSHHHHHHENLYFQG) were added to the 5’ end of the insert sequence. Recombinant bacmid was generated according to the manufacturer’s instructions, and successful transposition was confirmed by PCR.

**cGAS expression.** *Spodoptera frugiperda* (Sf-9) cells were cultured in SF-900-III SFM medium (Invitrogen #12658-027) in Corning 3 L baffled flasks (#431253, polycarbonate, vented cap) at 65 rpm, on a Lab Companion shaker (SKC6200, 2 inch orbit) incubated a 27^o^ C in a Thermo Scientific Incubator (Model #3940).

A 1ml aliquot of BIIC*^1^* [baculovirus infected insect cells preserved at 1x10^7^ vc/ml in freezing medium: 90% (v/v) SF-900-III SFM supplemented with 10gl^-1^ (w/v) bovine serum albumin (Sigma-Aldrich) and 10% (v/v) DMSO (Sigma-Aldrich)] was removed from -80 ^o^C preservation, rapidly thawed and diluted 1 to 100 in SF-900-III media. One liter of log phase Sf-9 cells at a cell density of 1.75 x10^6^ cells/mL and >95% viability were infected with 5 ml per liter of BIIC suspension per 1x10^6^ viable cells/ml. Cell density, cell viability and cell diameter were determined daily using a ViCell analyzer (Beckman Coulter). The cells were harvested at 72 hours post infection when cell viability dropped to 77-83% and cell diameter increased ~2.75-3.3 μm. The resulting cell paste was collected by refrigerated centrifugation at 3,500g, aliquotted, flash frozen in liquid nitrogen and stored at -80 ^o^C.

**cGAS Purification.** The purification protocol was based on Kranzusch et al.*^2^* with modifications. Briefly, cells (~50 g) were lysed in buffer A (20 mM Hepes pH 7.5, 400 mM NaCl, 10% Glycerol, 1 mM TCEP) with benzonase (Sigma), using a microfluidizer with 3 passes at 18K psi. The solution was clarified by centrifugation at 10,000 g for 30 min. The supernatant was applied to 5 x 5 ml HisTrap FF crude columns in tandem pre-equilibrated in Buffer A. The column was washed with buffer A with 50 mM imidazole, buffer A with 1 M NaCl, and then eluted with buffer A with 300 mM imidazole.

The eluted fractions were pooled, diluted 1:1 with Buffer C (20 mM Hepes pH 7.5, 10% Glycerol, 1 mM TCEP) and half the HisTrap eluate was loaded onto 2 x 5 ml Heparin columns pre-equilibrated in buffer C. The column was washed with buffer C with 500 mM NaCl and eluted with a gradient from 500 mM to 1 M NaCl. cGAS eluted in two peaks, Fraction #1 and Fraction#2. The heparin chromatography was repeated with the second half of the HisTrap eluate. Fraction #1 pools were combined as were fraction#2 pools. Each peak pool was loaded on a S200 26/60 equilibrated in 20 mM Hepes pH 7.5, 250 mM KCl, 1 mM TCEP, 10% glycerol. The S200 pools from each fraction were combined, concentrated to 1 mg/ml, aliquoted and flash frozen in liquid nitrogen. Mass spec analysis confirmed that Fraction #1 and Fraction #2 had the expected identical mass. Fraction #2 was routinely used for cGAS biochemical assays. cGAS enzyme with the His tag had more activity than cGAS with the His tag removed by TEV cleavage so all preparations retained the His tag.

All procedures were conducted at 4 ^o^ C. All buffers contained 1X ROCHE COMPLETE^TM^ proteinase inhibitors EDTA-free and benzamidine. The final storage buffer did not contain benzamidine.

**Cellular Assays.** THP-1 Dual monocytes cells containing NF-κB-SEAP and IRF-Luc Reporter (InvivoGen cat # thpd-nfis) were plated at 1x10^5 cells/well in U bottom 96 well plates in 100μL RPMI media containing10% FBS. Tested compounds were diluted in 100% DMSO to stock concentration of 30 mM. The TBK1 inhibitor BX-795 was obtained from Sigma-Aldrich (cat#SML0694-5MG) and used as a positive control. Cells were pretreated with DMSO or compounds for 1 hr at room temperature, followed by Lipofectamine™ 2000 (Thermo Fisher cat# 11668027) transfection with salmon sperm DNA (InvivoGen cat# tlrl-sdef) at 100 ng/well. Cells were incubated for 12 hrs at 37 ^◦^C in a 5% CO_2_ incubator followed by centrifugation at 1000 rpm for 5 min. Media was collected and Lucia luciferase was measured using Quanti-Luc substrate (InvivoGen cat# rep-qlc1) according to the manufacturer’s protocol. Viability of cells was assessed using Promega CellTiter Glo luminescent viability assay (Promega cat# G7570) according to the manufacturer’s protocol. All data points were normalized to the salmon sperm DNA-treated wells (without compound) and expressed as percent of signal.

**Nuclear Magnetic Resonance (NMR).** All NMR spectra were collected on a Bruker Avance III series 600 MHz spectrometer equipped with a 1.7 mm micro cryoprobe. For fragment screening, the Pfizer fragment library (~3,200 fragments) was screened against 3 μM truncated h-cGAS in 20 mM hepes (pH 7.5) buffer containing 150 mM KCl and 1 mM TCEP, and no DNA. The concentration of each fragment was 300 µM, and each sample contains either five or ten fragments in the mixture. Saturation transfer difference (STD) spectra for each fragment mixture were collected and binders were identified by comparing their STD signals with 1D reference spectra of the fragments in the mixture. For cGAMP competition binding experiments, the STD spectra of the samples containing both the compound of interest and 200 µM cGAMP were collected at the aforementioned protein and buffer condition, and the STD signal strength of the cGAMP was compared to a reference STD spectra that contains only the 200 µM cGAMP. All spectra were collected at 25 ^o^C. The affinity of compound binding at the active site was estimated based on the percentage change of cGAMP STD signal using the competition binding equation with the K_D_ value of cGAMP of 190 µM.

**SPR assays.** SPR experiments were performed using NTA sensor chips in a BIACORE T200 (GE Healthcare). cGAS was immobilized to a final density *ca* 5000-10000 RU. Sample-dependent responses were subtracted from channel 1, which had neither Ni or protein immobilized on it. Data were further corrected by subtracting a zero-concentratio blank from all compounds. Compounds were injected for 30-60 s, dissociation was measured for 30-120 s at 60 μL/min, 4 °C. Experiments were performed in buffer contained 20 mM HEPES (pH 7.5), 150 mM KCl, 20 mM MgCl_2_, 1 mM TCEP and 1% DMSO. Samples were injected with a top concentration, followed by 2-fold dilutions, and a final blank over 12 injection points. For binding assays the top concentration was 600 μM for compounds **15** and **16**; 200 μM for compound **20**, and 100 μM for **PF-06928215**. Dissociation equilibrium constants (K_D_) were determined using a binary association model (T200 BIA Eval software).

**Structure determination.** Crystals of cGAS_161_ were grown using conditions similar to a previous report*^3^*. Protein was concentrated to 4-6 mg/ml, and then mixed at a 2:1 protein to precipitate in a sitting drop format. Crystalization was against a 65 μL well containg PEG 3350 (18-20% v/v), 0.2 M ammonium citrate (pH 7) at 4 °C. Rod-shaped crystals grew to their final size within 5-7 days. Compound density was significantly reduced when freezing with low molecular weight poly ethylene glycol as cryoprotectants (*eg* ethylene glycol or PEG with average molecule weights less than 2000 Da). Our cryoprotectant was made using a final concentration of 23% PEG 3350, 0.2 M ammonium citrate (pH 7), which generally allowed for distinct compound density. Solid compounds were dissolved into cryoprotectant to 50 mM, soaks were performed at 4 °C for *ca* 5-10 min. Crystals were flash frozen in liquid nitrogen and data collected at the Argonne National Lab (IMCA) beamline. PDB codes for cGAS in complex with compound 15, 16, 20 and PF-06928215 are 5V8O, 5V8H, 5V8J and 5V8N. Refinement statistics are presented in SI Table 1.

**Synthesis of cGAMP derivatives**

**General**. All reactions were performed in glassware under a positive pressure of nitrogen. Guanosine (N-*i*-Bu) 3'-TBS CED phosphoramidite **6** (Part No. ANP-5683) was purchased from ChemGenes and used without further purification. All other chemicals were obtained from Aldrich or Fisher and used as received, unless noted otherwise. Solvents were commercial anhydrous grade and used as received. ^1^H NMR spectra were recorded on a Bruker spectrometer at 400 MHz or 500 MHz. The chemical shifts were reported relative to residual chloroform (7.26 ppm) or acetonitrile (1.94 ppm). Analytical thin layer chromatography (TLC) was performed on 60 F254 glass plates pre-coated with a 0.25-mm thickness of silica gel purchased from EMD chemical Inc. and TLC plates were visualized with UV light. Column chromatography was performed on TELEDYNE ISCO device.

**(2R,3S,4R,5R)-2-((bis(4-methoxyphenyl)(phenyl)methoxy)methyl)-5-(6-chloro-9H-purin-9-yl)tetrahydrofuran-3,4-diol (2)**. To a solution of 6-chloro-9-β-*D*-(−)-ribofuranoside (**1**) (1.0 g, 3.49 mmol) in anhydrous pyridine (20 mL) was added a solution of 4,4’-dimethoxytrityl chloride (1.77 g, 5.23 mmol) in anhydrous pyridine (10 mL). The resulting solution was stirred at ambient temperature for 3 h. Upon completion, the reaction was concentrated in vacuo and the crude was purified by column chromatography using silica gel (0 to 10% MeOH in DCM) to provide the desired product **2** as a colorless glassy solid (1.55 g, 75%). ^1^H NMR (500MHz, METHANOL-d4) δ 8.64 (d, J=9.0 Hz, 2H), 8.58 - 8.50 (m, 2H), 7.50 - 7.35 (m, 3H), 7.33 - 7.25 (m, 3H), 7.25 - 7.12 (m, 3H), 6.85 - 6.72 (m, 4H), 6.14 (d, J=4.9 Hz, 1H), 4.97 (t, J=4.9 Hz, 1H), 4.52 (t, J=4.9 Hz, 1H), 4.26 (q, J=4.4 Hz, 1H), 3.77 (d, J=1.7 Hz, 6H), 3.48 - 3.37 (m, 2H). LCMS (ESI), m/z: calculated for C_31_H_30_ClN_4_O_6_ [M+H]^+^ 589.2, found 589.1.

***Tert*-butyl (2-((9-((2R,3R,4S,5R)-5-((bis(4-methoxyphenyl)(phenyl)methoxy)methyl)-3,4-dihydroxytetrahydrofuran-2-yl)-9H-purin-6-yl)amino)ethyl)carbamate (3)**. To a solution of **2** (400 mg, 0.68 mmol) in ethanol (6.8 mL) was added 1-*N*-Boc-aminoethylenediamine (141 mg, 0.88 mmol) and trimethylamine (30 µL, 22 mg, 0.21 mmol). The resulting solution was stirred at ambient temperature overnight. The reaction was concentrated in vacuo and the crude was purified by column chromatography using silica gel (0 to 10% MeOH in DCM) to provide the desired product **3** as a colorless glassy solid (295 mg, 61%). ^1^H NMR (500MHz, METHANOL-d4) δ 8.20 (s, 2H), 7.44 - 7.37 (m, 2H), 7.32 - 7.26 (m, 4H), 7.25 - 7.13 (m, 3H), 6.85 - 6.74 (m, 4H), 6.04 (d, J=4.4 Hz, 1H), 4.81 (t, J=4.8 Hz, 1H), 4.48 (t, J=4.9 Hz, 1H), 4.26 - 4.17 (m, 1H), 3.75 (s, 6H), 3.69 (br. s., 2H), 3.45 - 3.33 (m, 4H), 1.38 (s, 9H). LCMS (ESI), m/z: calculated for C_38_H_45_N_6_O_8_ [M+H]^+^ 713.3, found 713.0.

***Tert*-butyl (2-((9-((2R,3R,4R,5R)-5-((bis(4-methoxyphenyl)(phenyl)methoxy)methyl)-3-((tert-butyldimethylsilyl)oxy)-4-hydroxytetrahydrofuran-2-yl)-9H-purin-6-yl)amino)ethyl)-carbamate (4)**. To a solution of **3** (315 mg, 0.44 mmol) and imidazole (90 mg, 1.33 mmol) in anhydrous pyridine (4.4 mL) was added TBSCl (100 mg, 0.66 mmol). The resulting solution was stirred at ambient temperature overnight. The reaction was concentrated in vacuo. The crude was taken up in DCM (80 mL), and washed with H_2_O (x3) and brine, dried over Na_2_SO_4_, filtered and concentrated in vacuo. The crude was purified by column chromatography using silica gel (10 to 100% EtOAc in heptanes) to provide the desired product **4** as a white solid (139 mg, 38%) and the undesired regioisomer 4a as a white solid (173 mg, 47%). The undesired regioisomer **4a** could be converted to **4** in 46% yield by stirring in MeOH in the presence of 0.1% Et3N. Spectroscopic data for **4**: ^1^H NMR (500MHz, DMSO-d6) δ 8.26 (s, 1H), 8.15 (br. s., 1H), 7.77 (br. s., 1H), 7.44 - 7.35 (m, 2H), 7.30 - 7.17 (m, 6H), 6.91 - 6.82 (m, 4H), 5.95 (d, J=4.9 Hz, 1H), 5.11 (d, J=5.9 Hz, 1H), 4.83 (t, J=4.9 Hz, 1H), 4.25 (d, J=5.4 Hz, 1H), 4.09 (q, J=4.5 Hz, 1H), 3.73 (s, 6H), 3.51 (br. s., 2H), 3.30 - 3.21 (m, 2H), 3.17 (q, J=6.1 Hz, 2H), 1.35 (s, 9H), 0.76 (s, 9H), -0.04 (s, 3H), -0.13 (s, 3H). LCMS (ESI), m/z: calculated for C_44_H_58_N_6_O_8_Si [M+H]^+^ 827.4, found 827.2. Spectroscopic data for **4a**: ^1^H NMR (500MHz, DMSO-d6) δ 8.30 (s, 1H), 8.14 (br. s., 1H), 7.76 (br. s., 1H), 7.34 (d, J=7.3 Hz, 2H), 7.29 - 7.15 (m, 6H), 6.88 (br. s., 1H), 6.86 - 6.76 (m, 4H), 5.89 (d, J=5.1 Hz, 1H), 5.38 (d, J=6.1 Hz, 1H), 4.84 (q, J=5.4 Hz, 1H), 4.53 - 4.43 (m, 1H), 4.08 - 3.97 (m, 1H), 3.72 (s, 6H), 3.51 (br. s., 2H), 3.35 (dd, J=4.4, 10.5 Hz, 1H), 3.16 (q, J=5.8 Hz, 2H), 3.11 (dd, J=4.9, 10.5 Hz, 1H), 1.36 (s, 9H), 0.83 (s, 9H), 0.07 (s, 3H), 0.03 (s, 3H). LCMS (ESI), m/z: calculated for C_44_H_59_N_6_O_8_Si [M+H]^+^ 827.4, found 827.2.

***Tert*-butyl (2-((9-((2R,3R,4R,5R)-5-((bis(4-methoxyphenyl)(phenyl)methoxy)methyl)-3-((tert-butyldimethylsilyl)oxy)-4-(((2-cyanoethoxy)(diisopropylamino)phosphanyl)oxy)-tetrahydrofuran-2-yl)-9H-purin-6-yl)amino)ethyl)carbamate (5)**. To a solution of **4** (195 mg, 0.24 mmol) in THF (1.6 mL) was added 1-N-methyl-1H-imidazole (9.7 mg, 0.12 mmol) and 2,4,6-trimethylpyridine (200 mg, 218 μL, 1.65 mmol), and then 2-cyanoethyl-N,N-diisopropylchlorophosphoramidite (167 mg, 0.158 mL, 0.71 mmol) was added dropwise via a syringe. The reaction turned cloudy within 30 sec and was stirred at ambient temperature. After 2 h, the reaction mixture was diluted with EtOAc, washed with saturated NaHCO_3_ solution and brine, dried over Na_2_SO_4_, filtered and concentrated in vacuo to give a colorless oil. The crude was purified by column chromatography using silica gel (10 to 60% EtOAc in heptanes with 1% Et_3_N) to provide the desired product **5** as an inseparable mixture of two phosphoramidite diastereomers (165 mg, 68%, white solids). ^1^H NMR (400MHz, CD_3_CN) δ 8.20 – 8.12 (m, 1H), 8.05 – 7.96 (m, 1H), 7.52 – 7.44 (m, 2H), 7.42 - 7.15 (m, 7H), 6.91 – 6.79 (m, 4H), 6.63 – 6.37 (m, 1H), 5.99 – 5.88 (m, 1H), 5.64 – 5.50 (m, 1H), 5.21 – 5.07 (m, 1H), 4.55 – 4.43 (m, 1H), 4.43 - 4.26 (m, 1H), 3.76 (s, 6H), 3.72 – 3.52 (m, 6H), 3.52 – 3.43 (m, 1H), 3.36 – 3.25 (m, 3H), 2.49 – 2.39 (m, 2H), 1.36 (s, 9H), 1.21 – 1.12 (m, 12H), 0.77 (s, 9H), -0.01 – -0.10 (m, 3H), -0.17 – -0.22 (m, 3H). LCMS (ESI), m/z: calculated for C_53_H_76_N_8_O_9_PSi [M-N(*i*-Pr)_2_+H_2_O]^+^ 944.4, found 944.2.

**Synthesis of Boc-ethylenediamine-2’,3’-cGAMP (10)**

To a solution of guanosine (N-*i*-Bu) 3'-TBS CED phosphoramidite **6** (237 mg, 0.24 mmol) in acetonitrile (1.22 mL) and H_2_O (8.8 μL, 0.49 mmol) was added pyridinium trifluoroacetate (56.6 mg, 0.29 mmol). The resulting colorless solution was stirred for 1 min, and then t-BuNH_2_ (1.22 mL) was added. After stirring for 10 min, the solvent was removed in vacuo, and the residue was azeotroped with acetronitrile (x3) to give a white foam solid. This intermediate was dissolved in DCM (2.94 mL) and H_2_O (44 μL, 2.44 mmol), and then a solution of dichloroacetic acid (6% in DCM, 177 μL, 2.2 mmol) was added as the reaction turned bright red. After stirring for 10 min, pyridine (340 mg, 0.35 mL, 4.3 mmol) was added slowly as the reaction turned colorless. The reaction was concentrated in vacuo, and the residue was azeotroped with acetonitrile (x3) to give a pink semi-solid. The crude was used in the next step without further purification.

To a solution of **7** (130 mg, 0.24 mmol) in acetonitrile (1.6 mL) was added adenosine **5** (326 mg, 0.32 mmol). The reaction mixture turned translucent. After stirring for 2 min, tert-butyl hydroperoxide (66 mg, 0.133 mL, 0.73 mmol) was added as the reaction turned slightly pink. The reaction was stirred at ambient temperature for 30 min, and then cooled down to 0 ^o^C and quenched with 33% aqueous NaHSO_3_ solution (0.122 mL). The ice bath was removed and the reaction was stirred at ambient temperature for 5 min. The solvent was removed in vacuo to afford **8** as a viscous yellow oil, which was used in the next step without further purification.

To a yellow solution of **8** (359 mg, 0.24 mmol) in DCM (4.0 mL) and H_2_O (44 mg, 44 μL, 2.4 mmol) was added a solution of dichloroacetic acid (276 mg) in DCM (6 wt% solution, 4.0 mL). The resulting orange solution was stirred for 10 min, and then quenched with pyridine (2.5 mL) to give a pale yellow solution. The reaction was concentrated to a small volume, and then additional pyridine (7.5 mL) was added and concentrated to ~5.0 mL. To this solution was added 2-chloro-5,5-dimethyl-1,3,2-dioxaphosphorinane 2-oxide (DMOCP) (158 mg, 0.85 mmol). After stirring for 10 min, H_2_O (154 mg, 0.154 mL, 8.5 mmol) was added followed immediately by iodine (81 mg, 0.32 mmol). The resulting brown solution was stirred for 5 min, and then poured into a 0.14 wt% aqueous solution of NaHSO_3_ (34 mL) to give a white slurry, which slowly turned pale translucent yellow. After stirring this mixture for 5 min, solid NaHCO_3_ (984 mg, 11.7 mmol) was added slowly as H_2_ gas was evolved. The mixture was extracted with EtOAc/Et_2_O (v/v = 1/1, 50 mL x 2). The organic extracts were combined and concentrated in vacuo, and azeotroped with toluene (3 mL x 3) to afford **9** as a viscous yellow oil. The crude was used in the next step without further purification.

To a solution of **9** (285 mg, 0.24 mmol) in acetonitrile (1.2 mL) was added tert-butylamine (1.22 mL). The resulting solution was stirred for 10 min, and then concentrated in vacuo. The residue was azeotroped with acetonitrile (x2) to give a pale yellow form solid. To this intermediate was added methylamine (33% in EtOH, 12 mL), and the resulting pale yellow solution was stirred for 1.5 h. The reaction was concentrated in vacuo, and the crude was taken up in Et_3_N and then concentrated in vacuo to give a yellow semi-solid. To this intermediate was added Et_3_N•3HF (1.96 g, 2.0 mL, 12.2 mmol). The resulting mixture was stirred at ambient temperature overnight. The reaction was quenched with 1.0 M aqueous solution of NH_4_OAc (1.88 g, 24 mL, 24.4 mmol) and heated at 35 ^o^C for 30 min. After cooling down to ambient temperature, the reaction mixture was filtered and the solids were rinsed with a small amount of H_2_O. The yellow filtrate was concentrated to a small volume and purified directly using reverse phase HPLC eluting with 2% MeCN w. 0.02% TFA to 27% MeCN w. 0.02% TFA in H_2_O over a 20-min gradient. The fractions were combined and dried in a lyophilizer to provide the desired product **10** (retention time = 14.5 min) as a fluffy white solid (29.2 mg, 14.7% over 11 steps). ^1^H NMR (500MHz, D_2_O, 30 mM Na_2_HPO_4_/NaH_2_PO_4_, pH 7.4, c = 2.5 mM, 25 °C) δ 8.21 - 8.33 (m, 2H), 7.85 (s, 1H), 6.11 - 6.17 (m, 1H), 5.93 (d, J=8.3 Hz, 1H), 5.62 (br. s., 1H), 5.04 (br. s., 1H), 5.01 - 4.70 (m, 1H), 4.59 (d, J=3.9 Hz, 1H), 4.46 (d, J=10.8 Hz, 2H), 4.39 (br. s., 1H), 4.20 - 4.26 (m, 1H), 4.10 - 4.18 (m, 2H), 3.78 - 3.85 (m, 1H), 3.52 - 3.60 (m, 1H), 3.42 (br. s., 1H), 3.35 (br. s., 1H), 1.13 - 1.34 (m, 5H), 1.04 ppm (br. s., 4H). ^31^P NMR (162MHz, D_2_O, 30 mM Na_2_HPO_4_/NaH_2_PO_4_, pH 7.4, sodium phosphate used as a reference and set to 3.00 ppm, c = 2.5 mM, 50 °C) δ 1.11, 0.15. LCMS (ESI), m/z: calculated for C_27_H_38_N_11_O_15_P_2_ [M+H]^+^ 818.2, found 818.5.

**Synthesis of Ethylenediamine-2’,3’-cGAMP (11)**

A solution of **10** (27 mg, 0.033 mmol) in 5% TFA in H_2_O (2.7 mL) was stirred at ambient temperature for 24 h. Upon completion, the reaction was dried in a lyophilizer to afford the desired product **11** as a TFA salt (24 mg, 87%). The product was used directly without further purification. LCMS (ESI), m/z: calculated for C_22_H_30_N_11_O_13_P_2_ [M+H]^+^ 718.2, found 718.4.

**Synthesis of NHS-C6-Ethylenediamine-2’,3’-cGAMP (12)**

To a solution of NHS-C6-NHS (7.8 mg, 0.021 mmol) and DIPEA (5.4 mg, 7.3 μL, 0.042 mmol) in DMSO (0.2 mL) was added dropwise a solution of **11** (3.5 mg, 0.0042 mmol) in DMSO (0.2 mL). The resulting clear solution was stirred for 30 min. The crude reaction mixture was purified directly using reverse phase HPLC eluting with 5% MeCN w. 0.1% formic acid to 40% MeCN w. 0.1% formic acid in H_2_O over a 25-min gradient. The fractions were combined and dried in a lyophilizer to provide the desired product **12** (retention time = 20 min) as a fluffy white solid (3.4 mg, 83%). This compound existed as an inseparable mixture of diastereomers based on NMR. ^1^H NMR (500MHz, D_2_O, 25 °C) δ 8.45 (s, 1H), 8.40 – 8.25 (m, 1H), 7.87 (s, 1H), 6.12 (s, 1H), 5.95 (d, *J* = 8.2 Hz, 1H), 5.70 – 5.60 (m, 1H), 5.10 – 5.00 (m, 1H), 4.85 – 4.65 (m, 5H), 4.61 (d, *J* = 3.9 Hz, 0.5H), 4.51 – 4.45 (m, 1H), 4.41 (s, 0.5H), 4.30 – 4.21 (m, 1H), 4.19 – 4.12 (m, 1H), 4.00 – 3.85 (m, 1H), 3.70 – 3.60 (m, 1H), 3.60 – 3.45 (m, 1H), 2.92 (s, 3H), 2.73 (s, 0.5H), 2.55 – 2.45 (m, 2H), 2.23 (s, 0.5H), 2.22 – 2.10 (m, 2H), 1.45 – 1.30 (m, 4H), 1.20 – 0.95 (m, 4H). LCMS (ESI), m/z: calculated for C_34_H_45_N_12_O_18_P_2_ [M+H]^+^ 971.2, found 971.7.

**Synthesis of NHS-PEG5-Ethylenediamine-2’,3’-cGAMP (13)**

To a solution of NHS-PEG5-NHS (11.2 mg, 0.021 mmol) and DIPEA (6.3 mg, 8.5 μL, 0.049 mmol) in DMSO (0.2 mL) was added dropwise a solution of **11** (3.5 mg, 0.0042 mmol) in DMSO (0.2 mL). The resulting clear solution was stirred for 30 min. The crude reaction mixture was purified directly using reverse phase HPLC eluting with 10% MeCN w. 0.1% formic acid to 40% MeCN w. 0.1% formic acid in H_2_O over a 25-min gradient. The fractions were combined and dried in a lyophilizer to provide the desired product **13** (retention time = 14 min) as a fluffy white solid (1.9 mg, 40%). ^1^H NMR (500MHz, D_2_O, 25 °C) δ 8.45 (s, 1H), 8.40 – 8.28 (m, 1H), 7.87 (s, 1H), 6.12 (s, 1H), 5.95 (d, *J* = 8.6 Hz, 1H), 5.70 – 5.60 (m, 1H), 5.10 – 5.01 (m, 1H), 4.90 – 4.65 (m, 4H), 4.61 (d, *J* = 3.9 Hz, 0.5H), 4.52 – 4.45 (m, 1H), 4.42 (s, 0.5H), 4.30 – 4.21 (m, 1H), 4.20 – 4.15 (m, 1H), 3.95 – 3.50 (m, 24H), 3.08 – 2.90 (m, 5H), 2.73 (s, 1H), 2.55 – 2.45 (m, 2H). LCMS (ESI), m/z: calculated for C_40_H_57_N_12_O_23_P_2_ [M+H]^+^ 1135.3, found 1135.8.

**Synthesis of NHS-C6-Ethylenediamine-2’,3’-cGAMP (14)**

To a solution of **11** (9.0 mg, 0.011 mmol) and DIPEA (14.0 mg, 18.9 μL, 0.11 mmol) in DMSO (1.0 mL) was added Biotin-PEG6-NHS (14.7 mg, 0.022 mmol). The resulting clear solution was stirred for 3 h. The crude reaction mixture was purified directly using reverse phase HPLC eluting with 5% MeCN w. 0.02%TFA to 22% MeCN w. 0.02% TFA in H_2_O over a 35-min gradient. The fractions were combined and dried in a lyophilizer to provide the desired product **14** (retention time = 24.5 min) as a fluffy white solid (8.6 mg, 62%). ^1^H NMR (500MHz, D_2_O, 50 °C) δ 8.93 (s,1H), 8.72 (br. s, 1H), 8.67 (s, 1H), 6.47 (s, 1H), 6.28 (d, *J* = 8.3 Hz, 1H), 5.82 (br. s, 1H), 5.21 (br. s, 1H), 5.03 – 4.97 (m, 1H), 4.82 – 4.70 (m, 12H), 4.68 – 4.63 (m, 1H), 4.63 – 4.59 (m, 1H), 4.52 – 4.45 (m, 1H), 4.42 – 4.38 (m, 1H), 4.35 – 4.30 (m, 1H), 4.10 – 3.75 (m, 20H), 3.60 – 3.55 (m, 2H), 3.53 – 3.48 (m, 1H), 3.18 (dd, *J* = 5.0, 13.0 Hz, 1H), 2.97 (d, *J* = 13.0 Hz, 1H), 2.74 (br. s, 1H), 2.45 (t, *J* = 7.3 Hz, 2H), 1.99 – 1.67 (m, 4H), 1.67 – 1.52 (m, 2H). LCMS (ESI), m/z: calculated for C_47_H_72_N_14_O_22_P_2_S [M+H]^+^ 1179.2, found 1179.9

**cGAMP-PPD**. To 1 mL 5 mg/mL PPD solution (in water) was added 0.1 mL 1 M NaHCO3, then 3.4 mg **Compound 13** (40 mg/mL in DMSO, 85 µL, 40 fold excess) was added slowly, and gently shaken at room temp for 90 minutes. The reaction solution was dialyzed (Slide-A-lyzer cassette, 7000 MWCO) against excess PBS for 48 hours at 4°C with multiple changes in PBS.

**cGAMP-Strepavidin.** To 0.5 ml 10 mg/ml streptavidin (in water) was added 1.15 mg **Compound 14** (40 mg/mL in DMSO) slowly, and the resulting solution was gently shaken at room temperature for 90 minutes. Excess compound and DMSO were removed by dialysis

(Slide-A-lyzer cassette, 7000 MWCO) against excess PBS for 48 hr at 4°C with multiple changes in PBS.

**cGAMP-BSA.** Imject BSA (Thermo Scientific) was dissolved in water to give 10 mg/mL BSA in 0.1 M sodium phosphate, 0.15 M NaCl, pH 7.2 with stabilizers. To 0.5 mL 10 mg/mL BSA solution, 3 mg **Compound 12** (40 mg/mL in DMSO) was added slowly and gently shaken at room temp for 90 minutes. Excess compound and DMSO were removed by dialysis (Slide-A-lyzer cassette, 7000 MWCO) against excess PBS for 48 hr at 4°C with multiple changes in PBS.

**cGAMP-Cy5.** cGAMP amine (**Compound 11**, 2.64 µmol, 44 mg/ml in water) was added to 2.1 mg (2.65 μmol) Cy5 mono NHS ester and 10 μL 2 M N,N-diisopropylethylamine in N-methyl-2-pyrrolidone (20 μmol). After 30 minutes, the reaction was purified directly by preparative high-performance liquid chromatography (HPLC). Preparative HPLC was performed using a Waters XBridge Peptide BEH C18 Prep Column, 130Å, 10 µm, 10 mm X 150 mm (Waters, part number 186003654), eluting with a linear slope gradient at 10 mL/min flow rate. Solvent gradient: Acetonitrile/water/trifluoroacetic acid (TFA) (12:88:0.1) to (51:49:0.1) over 40 min. Collected fractions were analyzed by analytical LCMS, and the fractions at 7.5-8.25 minutes judged as having adequate purity were pooled. ESMS: calculated m/z for desired (MH+) = 1356.53, found = 1356.36. The yield was determined by UV absorption at 648 nm (extinction coefficient = 250000), recovery was calculated as 1.34 mg (37%). The solution was aliquoted and evaporated in a vacuum centrifuge, and the product was stored at 4°C.

**General Procedure for Synthesis of cGAS inhibitors detailed in Table 1.** To a round bottom flask containing EDCI (2.0 equiv), 7-oxo-5-phenyl-4,7-dihydropyrazolo[1,5-a]pyrimidine-3-carboxylic acid (1.0 equiv), and HOBt (2.0 equiv) under nitrogen atmosphere was added DMF (0.10 M). The resulting mixture was allowed to stir at ambient temperature for 20 min, and *N*,*N*-diisopropylethylamine (4.0 equiv) and amine (1.5 equiv) were then added. The reaction was allowed to stir for 14 h, and was then concentrated *in vacuo* to afford the crude product. Subsequent purification by column chromatography provided the corresponding amides.

***N*-methyl-7-oxo-5-phenyl-4,7-dihydropyrazolo[1,5-a]pyrimidine-3-carboxamide (16)**

According to the general procedure, methylamine hydrochloride (265 mg, 3.92 mmol) was reacted to provide amide **16** as a white solid (447 mg, 85%). **^1^H NMR** (400 MHz, DMSO-d_6_): δ 8.48 (br s, 1H), 8.05-7.99 (m, 3H), 7.49-7.40 (m, 3H), 6.13 (s, 1H), 2.89 (s, 1H), ppm. **^13^C NMR** (100 MHz, DMSO-d6): (mix of OH and NH tautomers results in additional signals) δ 163.6, 163.5, 158.3, 157.2, 149.7, 142.1, 139.0, 128.9, 128.4, 126.6, 101.7, 101.6, 91.2, 25.2, 25.0 ppm. **LRMS** (ESI+) Calcd. for C_14_H_13_N_4_O_2_ (M+H): 269.1, Found: 269.1.

**N-(2-hydroxyethyl)-7-oxo-5-phenyl-4,7-dihydropyrazolo[1,5-a]pyrimidine-3-carboxamide (17)**

According to the general procedure, 2-aminoethan-1-ol (36 mg, 0.6 mmol) was reacted to provide amide **19** as a white solid (108 mg, 92%). **^1^H NMR** (400 MHz, DMSO-_d6_): δ 11.29 (br. s., 1 H), 8.53 (br. s., 1 H), 8.44 (s, 1 H), 7.84 (d, *J* = 6.02 Hz, 2 H), 7.66-7.57 (m, 3 H), 6.30 (s, 1 H), 3.49 - 3.58 (m, 2 H), 3.29 - 3.44 (m, 3 H) ppm. **^1^H NMR** (400 MHz, METHANOL-d_4_): δ ppm 8.37 (s, 1 H) 7.82 - 7.95 (m, 2 H), 7.56 - 7.71 (m, 3 H), 6.19 - 6.37 (m, 1 H), 3.74 (t, *J* = 5.77 Hz, 2 H), 3.49 - 3.58 (m, 2 H) ppm; **^13^C NMR** (100 MHz, DMSO-d_6_): δ 162.3, 155.7, 149.6, 143.0, 141.0, 131.4, 129.2, 126.9, 100.1, 96.3, 59.9, 41.4 ppm; **LRMS** (ESI+) Calcd. for C_15_H_14_N_4_O_3_ (M+H): 299.1, Found: 299.1.

**(7-oxo-5-phenyl-4,7-dihydropyrazolo[1,5-a]pyrimidine-3-carbonyl)glycine (18)**

According to the general procedure, *tert*-butyl glycinate (246 mg, 1.47 mmol) was reacted to provide the intermediate *tert*-butyl ester, which was then treated with TFA/DCM (1:1) for 15 h. The solvent was then removed *in vacuo* to afford the crude title compound as a residue, which was purified by RP-HPLC to afford pure title compound (**19**, 175 mg, 57%) as a white solid. **^1^H NMR** (400 MHz, DMSO-d_6_): δ 11.28 (br. s., 1 H), 8.90 (t, *J* = 5.77 Hz, 1 H), 7.82 (d, *J*=6.53 Hz, 2 H), 7.38 - 7.68 (m, 3 H), 6.30 (s, 1 H), 3.99 (br. s., 2 H) ppm; **^13^C NMR** (100 MHz, DMSO-d_6_): δ 171.3, 16.4, 155.7, 149.8, 143.2, 141.0, 131.3, 129.1, 127.0, 99.7, 96.6 ppm. **LRMS** (ESI+) Calcd. for C_15_H_12_N_4_O_4_ (M+H): 313.1, Found: 313.0.

**(S)-N-(1-hydroxypropan-2-yl)-7-oxo-5-phenyl-4,7-dihydropyrazolo[1,5-a]pyrimidine-3-carboxamide (19)**

According to the general procedure, (*S*)-2-aminopropan-1-ol (88 mg, 1.2 mmol) was reacted to provide amide **18** as a white solid (211 mg, 86%). **^1^H NMR** (400 MHz, DMSO-d6): δ 11.24 (br s, 1H), 8.46 (s, 1H), 8.17 (d, *J* = 7.4 Hz, 1H), 7.83 (d, *J* = 6.2 Hz, 2H), 7.60 (d, *J* = 7.0 Hz, 3H), 6.29 (s, 1 H), 4.08-4.01 (m, 1 H), 3.48 (dd, *J* = 5.9 Hz, 10.6 Hz, 1 H, 3.37 (dd, *J* = 6.3Hz, 10.6 Hz, 1 H), 1.16 (d, *J* = 6.6 Hz, 3H) ppm. **^13^C NMR** (100 MHz, DMSO-d6): δ 163.0, 161.8, 155.7, 149.5, 143.1, 141.0, 131.4, 129.2, 126.9, 100.1, 96.3, 64.4, 46.6, 17.2 ppm**LRMS** (ESI+) Calcd. for C_16_H_17_N_4_O_3_ (M+H): 313.1, Found: 313.0.

**(1*R*,2*S*)-2-(7-oxo-5-phenyl-4,7-dihydropyrazolo[1,5-a]pyrimidine-3 carboxamido) cyclohexane-1-carboxylic acid (PF-06928215)**

According to the general procedure, ethyl (1*R*,2*S*)-2-aminocyclohexane-1-carboxylate (248 mg, 1.5 mmol) was reacted to provide the intermediate ethyl ester, which was subsequently dissolved in 10 mL EtOH. A solution of 10% aqueous sodium hydroxide was added (3 mL) and the solution was stirred at room temperature for 15 h. The solution was then acidified to pH = 1 with 1 N HCl and the solvent was removed under reduced pressure to provide the crude title compound as a residue. The crude mixture was then purified via prep-HPLC to provide **PF-06928215** (78 mg, 14%) as a white solid. **^1^H NMR** (400 MHz, DMSO-d6): δ 11.30 (br s, 1H), 8.60 (s, 1H), 8.06 (br s, 1H), 7.83 (br s, 2H), 7.61 (br s, 3H), 6.29 (s, 1H), 4.38 (br s, 1H), 2.79 (br s, 1H), 1.98-1.89 (m, 2 H), 1.65-1.58 (m, 4H), 1.44-1.34 (m, 2H) ppm. **^13^C NMR** (100 MHz, DMSO-d6): δ 174.9, 163.2, 162.0, 156.2, 149.8, 143.7, 141.6, 131.8, 129.4, 127.2, 100.5, 96.6, 48.5, 44.1, 29.4, 25.0, 23.1, 22.3 ppm. **LRMS** (ESI+) Calcd. for C_20_H_21_N_4_O_4_ (M+H): 381.2, Found: 381.0.

***1H NMR and 13C NMR Spectra***


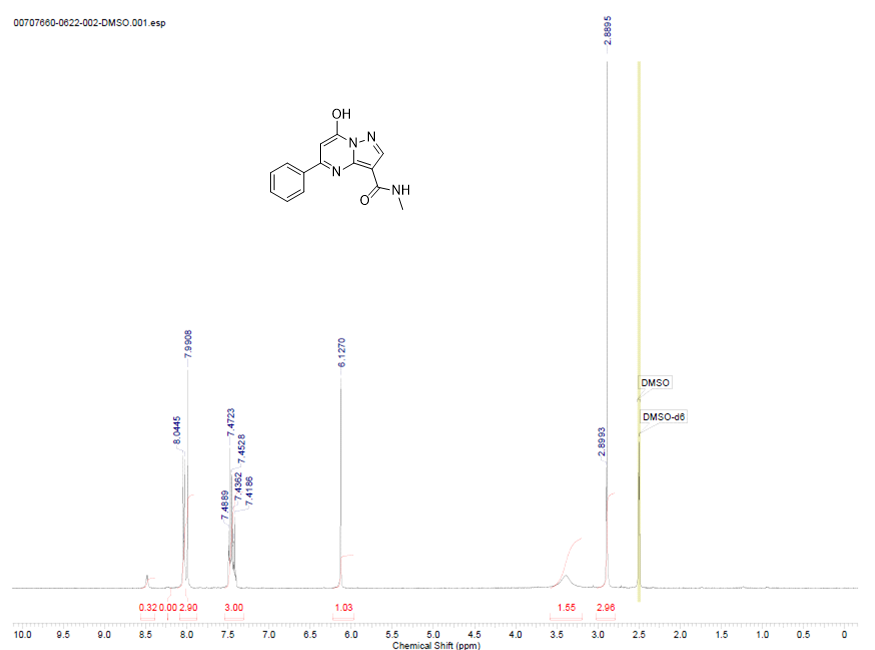


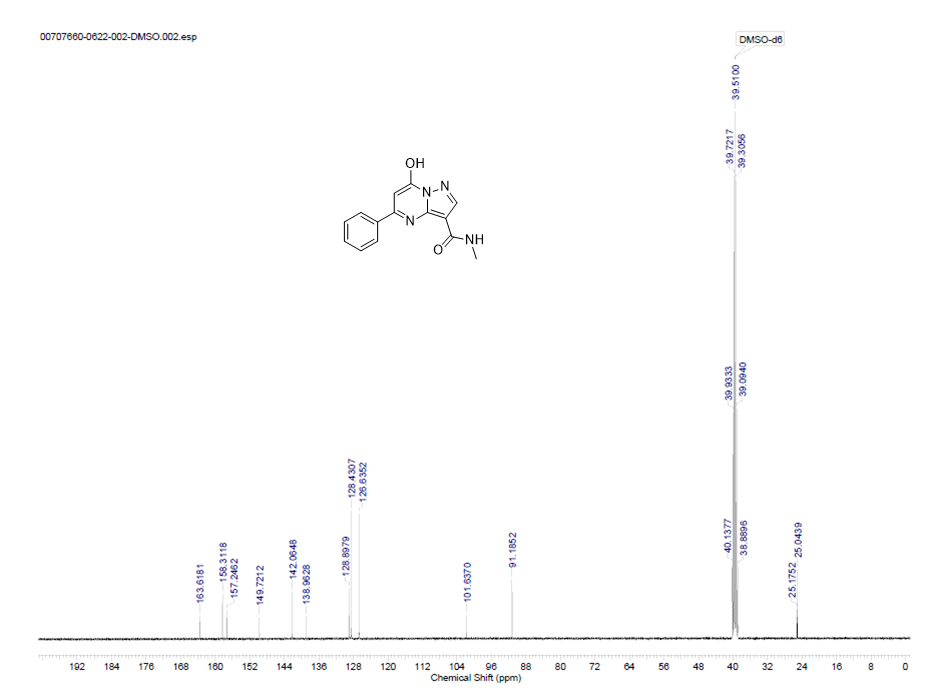

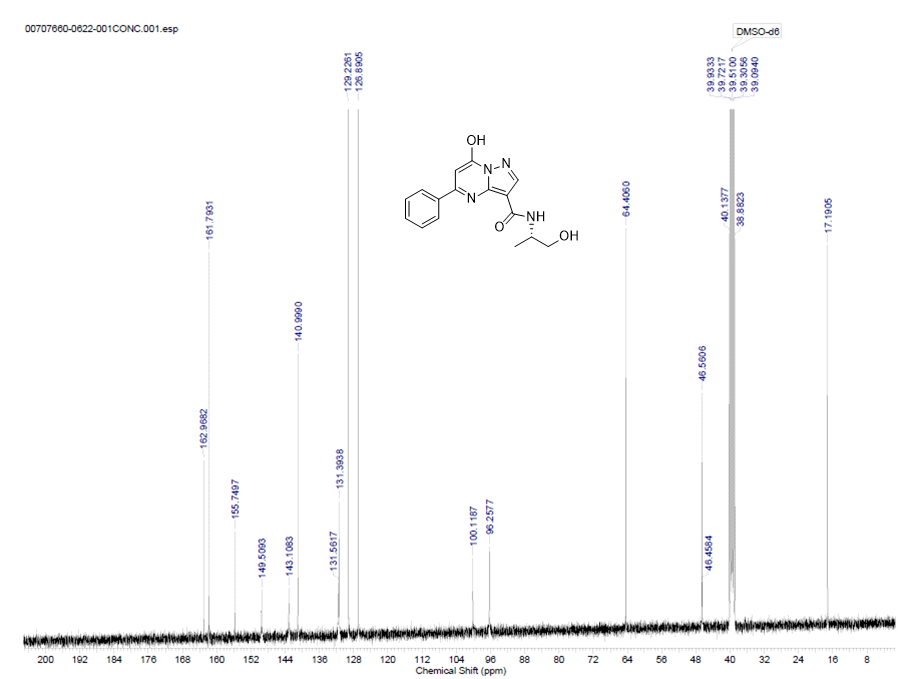


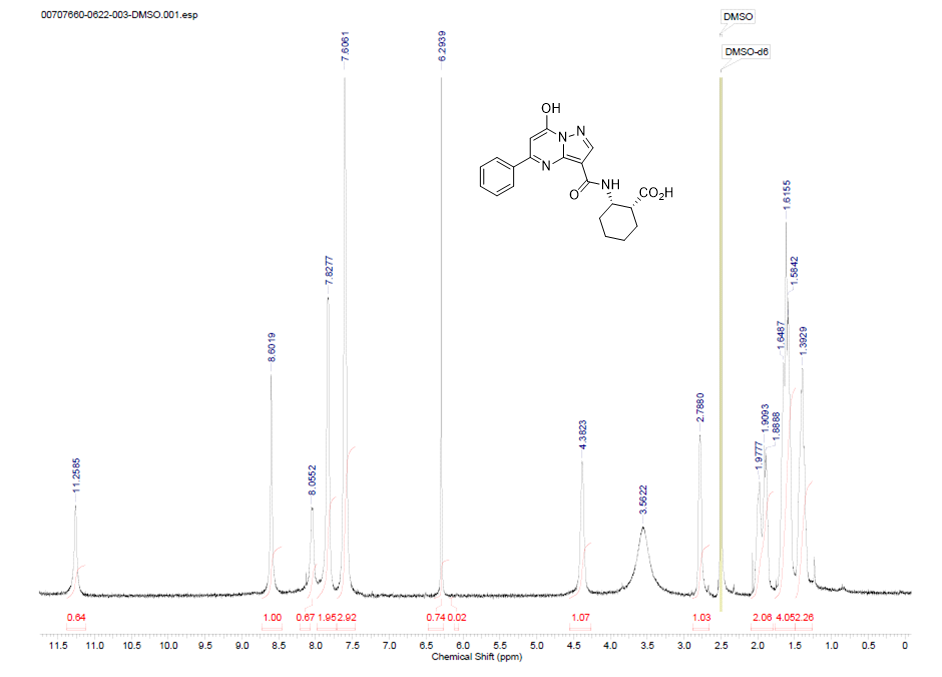


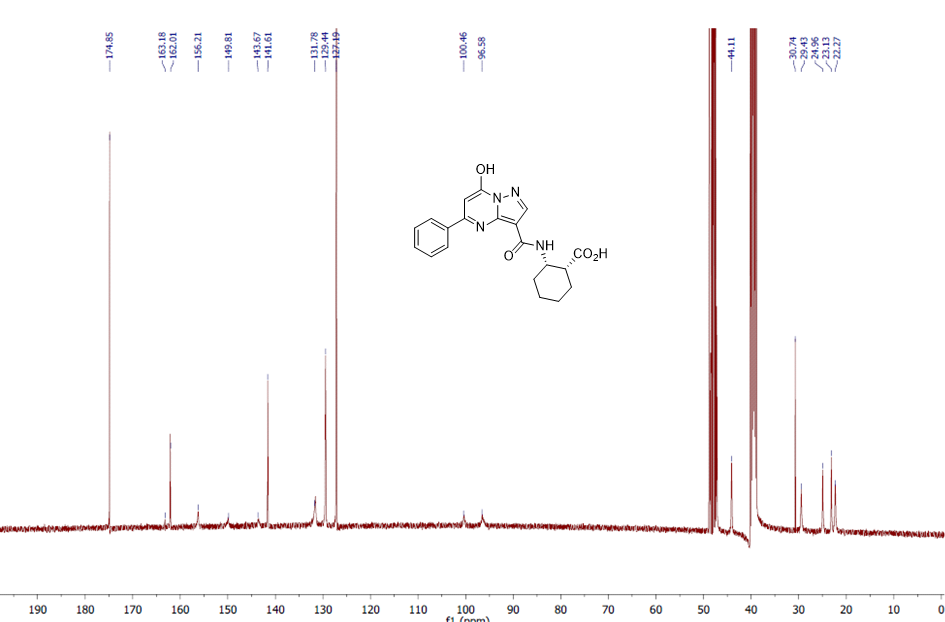


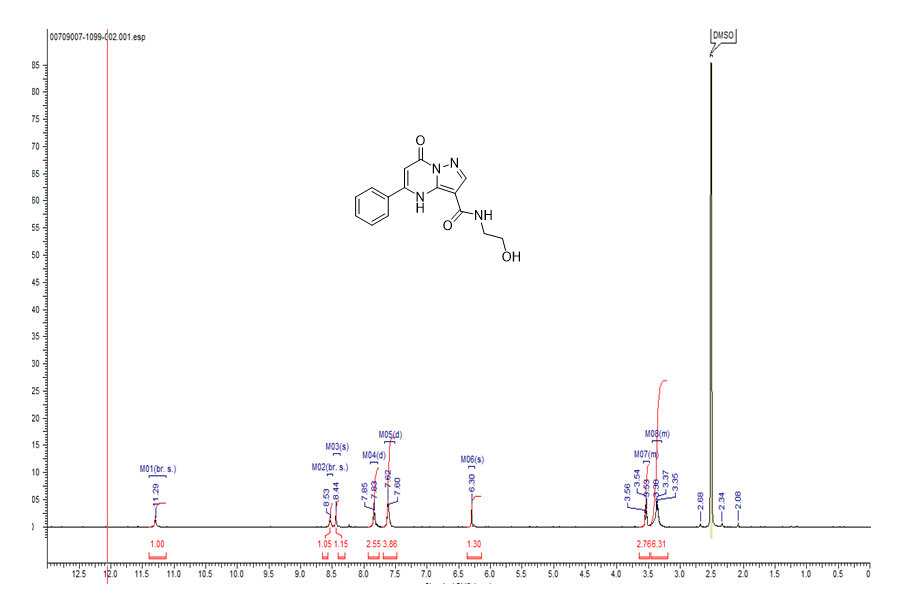


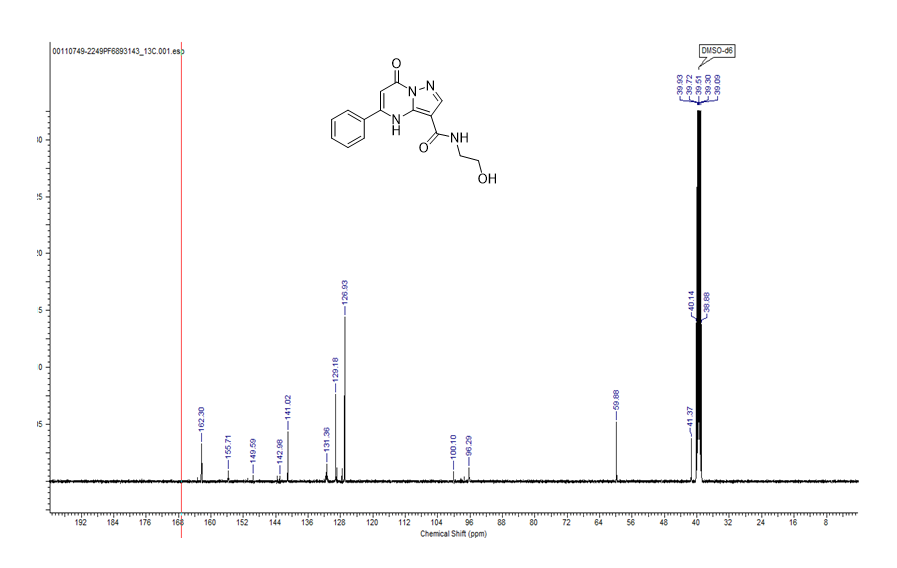


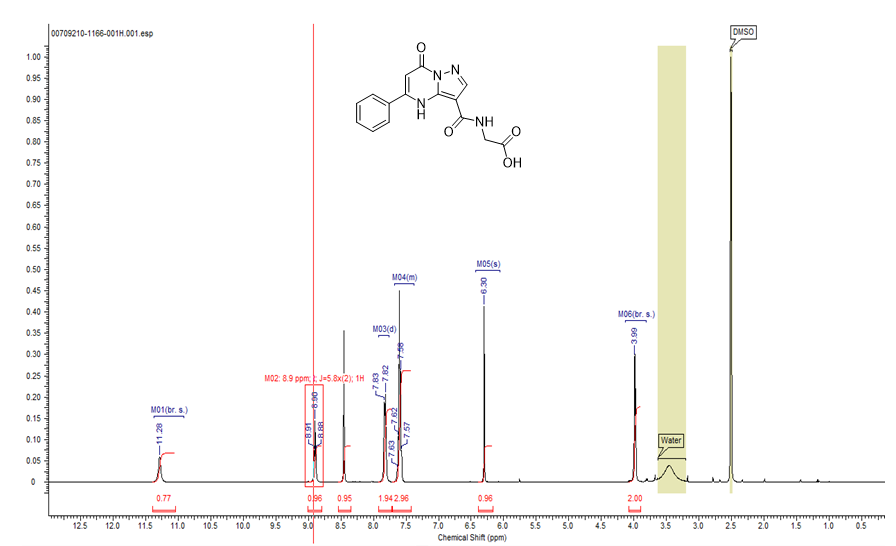


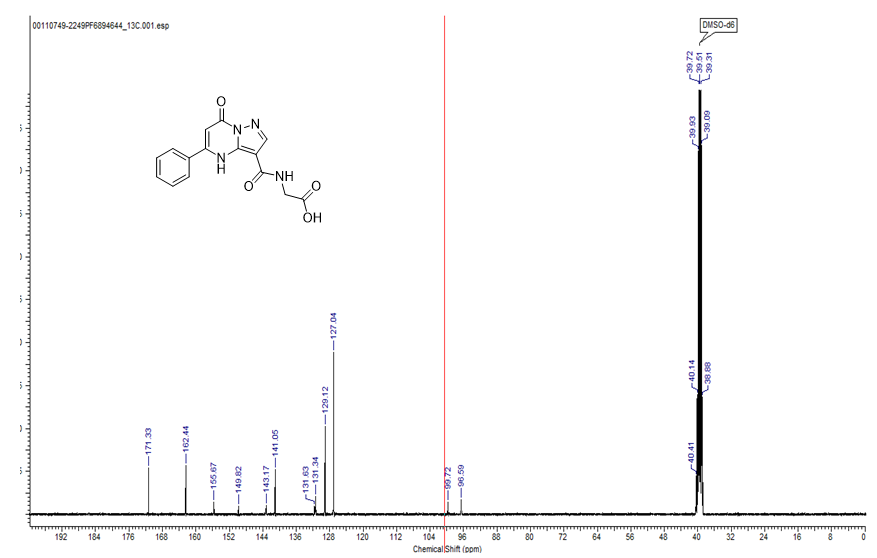


**Supplemental Figures**


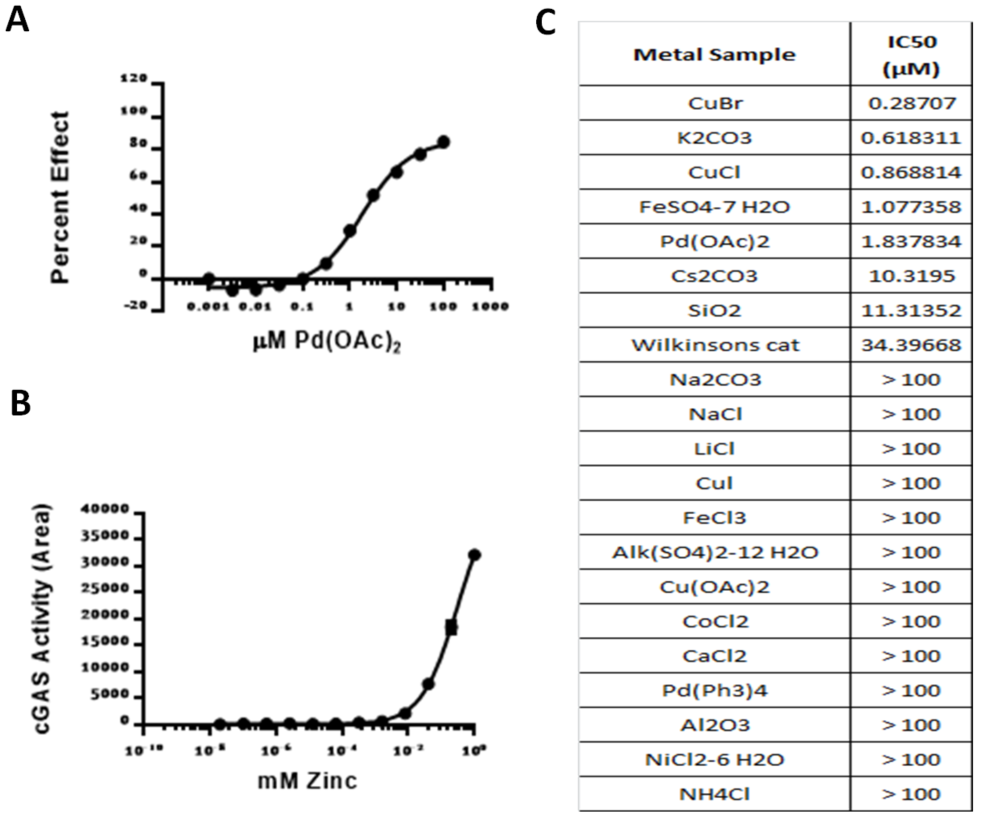


**S1 Figure. Modulation of cGAS enzyme activity by metals in the MS assay***.* (A) Inhibition dose response of Pd(OAc)_2_ with cGAS (1 nM); (B) Activation dose response of ZnCl_2_ with cGAS (1 nM) and ISD dsDNA (1nM); NB: lower activation multiples were observed with higher ISD DNA concentrations (data not shown); (C) Results from a panel of metal salts tested in the cGAS (1nM) assay in the presence of 1 nM ISD DNA.

**Supporting Information References**

[1] Wasilko, D. L., SE. (2006) TIPS: Titerless Infected Cells Preservation and Scale-Up, *BioProcessing J* *5*, 29-32.

[2] Kranzusch, P. J., Lee, A. S., Berger, J. M., and Doudna, J. A. (2013) Structure of human cGAS reveals a conserved family of second-messenger enzymes in innate immunity, *Cell reports* *3*, 1362-1368.

[3] Li, X., Shu, C., Yi, G., Chaton, C. T., Shelton, C. L., Diao, J., Zuo, X., Kao, C. C., Herr, A. B., and Li, P. (2013) Cyclic GMP-AMP synthase is activated by double-stranded DNA-induced oligomerization, *Immunity* *39*, 1019-1031.
