# Supplementary material for: Discovery of PF-06928215 as a high affinity inhibitor of cGAS enabled by a novel fluorescence polarization assay
Source: PLoS One. 2017 Sep 21;12(9):e0184843. doi: 10.1371/journal.pone.0184843 (PMC5608272; doi:10.1371/journal.pone.0184843)
Supplement: S2 Fig — (A) Apparent Km determination for GTP in the presence of 1 mM ATP; (B) Apparent Km determination for ATP in the presence of 0.3 mM GTP. (DOCX) [file pone.0184843.s002.docx]

**
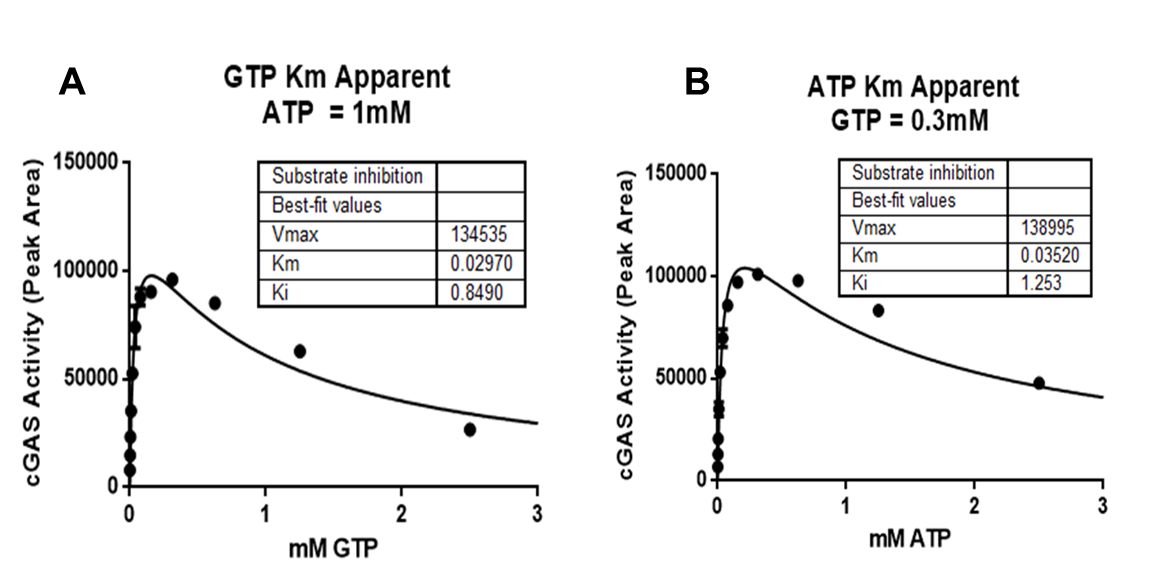
**

**S2 Figure. Nucleotide-dependence of cGAS enzyme activity.** (A) Apparent K_m_ determination for GTP in the presence of 1 mM ATP; (B) Apparent K_m_ determination for ATP in the presence of 0.3 mM GTP.
