# Supplementary material for: Discovery of PF-06928215 as a high affinity inhibitor of cGAS enabled by a novel fluorescence polarization assay
Source: PLoS One. 2017 Sep 21;12(9):e0184843. doi: 10.1371/journal.pone.0184843 (PMC5608272; doi:10.1371/journal.pone.0184843)
Supplement: S3 Fig — A protected and activated ethylenediamine adenosine analog was prepared (5) and coupled with a protected guanosine analog (7) in acetonitrile, followed by oxidation to the phosphate (8). Protecting group manipulation, cyclisation and further oxidation provided the protected cGAMP analog (9). Stepwise removal of the various protecting groups then provided the desired ethylenediamine-cGAMP analog (11). Compound 11 proved to be a highly useful intermediate, whereby the primary alkyl amino group could be selectively reacted with various linker groups, forming a stable amide bond, followed by subsequent conjugation or binding to various proteins for antibody generation or screening. (DOCX) [file pone.0184843.s003.docx]

**
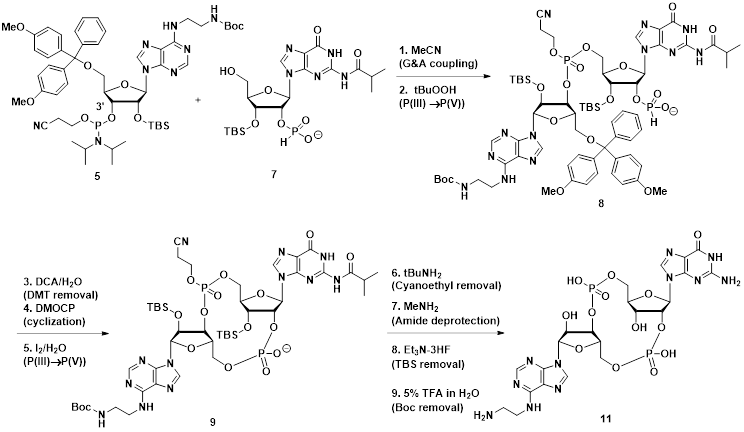
**

**S3 Figure. Preparation of a cGAMP analog containing an ethylenediamine functionality.** A protected and activated ethylenediamine adenosine analog was prepared (**5**) and coupled with a protected guanosine analog (**7**) in acetonitrile, followed by oxidation to the phosphate (**8**). Protecting group manipulation, cyclisation and further oxidation provided the protected cGAMP analog (**9**). Stepwise removal of the various protecting groups then provided the desired ethylenediamine-cGAMP analog (**11**). Compound **11** proved to be a highly useful intermediate, whereby the primary alkyl amino group could be selectively reacted with various linker groups, forming a stable amide bond, followed by subsequent conjugation or binding to various proteins for antibody generation or screening.
