# Supplementary material for: Discovery of PF-06928215 as a high affinity inhibitor of cGAS enabled by a novel fluorescence polarization assay
Source: PLoS One. 2017 Sep 21;12(9):e0184843. doi: 10.1371/journal.pone.0184843 (PMC5608272; doi:10.1371/journal.pone.0184843)
Supplement: S4 Fig — (A) An ethylenediamine-cGAMP analog linked through PEG5 to a reactive NHS ester (13) for subsequent attachment to PPD (cGAMP-PPD); (B) an ethylenediamine-cGAMP analog linked through PEG6 to a biotin molecule (14) for subsequent binding to streptavidin (cGAMP-strepavidin) were synthesized. Mice were immunized with a mixture of these protein conjugates. (C) serum was tested in a DELFIA immunoassay for reactivity against a further analog, ethylenediamine-cGAMP linked through C6 to a reactive NHS ester (12) which allowed conjugation to BSA (cGAMP-BSA). (D) an ethylenediamine-cGAMP analog conjugated to Cy5 was synthesized to be used as the fluorescently labelled cGAMP analogue in the FP assay. (DOCX) [file pone.0184843.s004.docx]

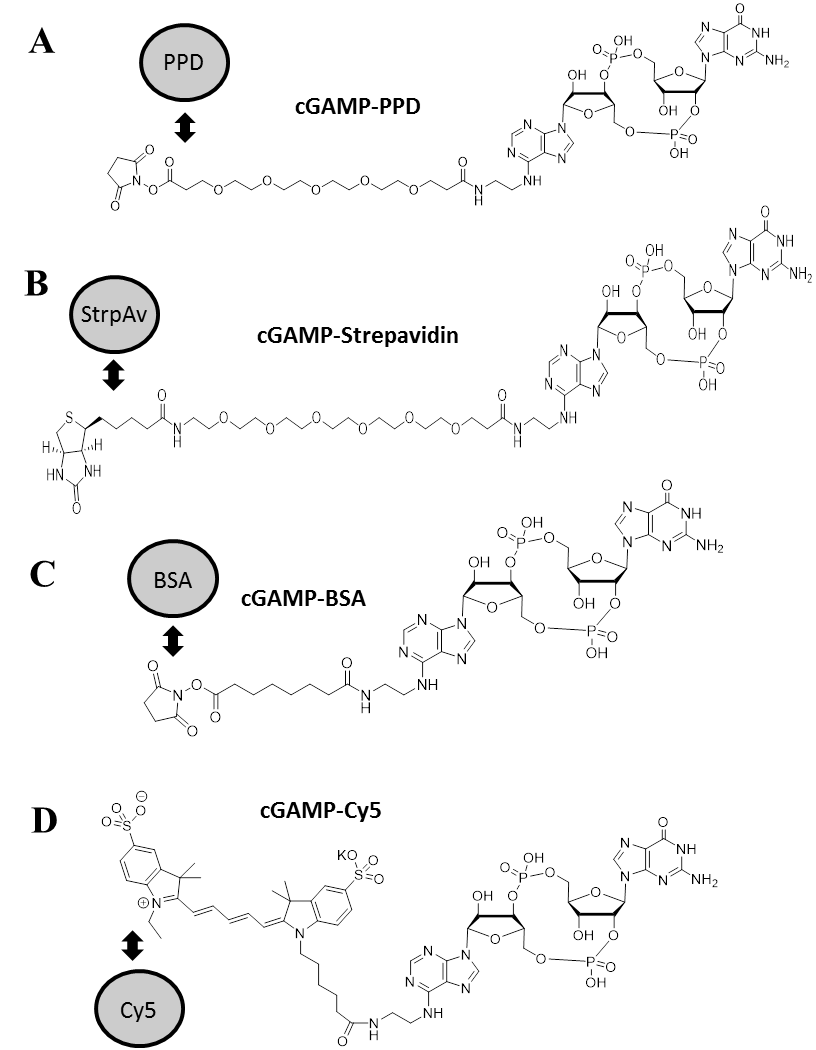


**S4 Figure. cGAMP derivatives for mAb production and screening***.* (A) An ethylenediamine-cGAMP analog linked through PEG5 to a reactive NHS ester (**13**) for subsequent attachment to PPD (cGAMP-PPD); (B) an ethylenediamine-cGAMP analog linked through PEG6 to a biotin molecule (**14**) for subsequent binding to streptavidin (cGAMP-strepavidin) were synthesized. Mice were immunized with a mixture of these protein conjugates. (C) serum was tested in a DELFIA immunoassay for reactivity against a further analog, ethylenediamine-cGAMP linked through C6 to a reactive NHS ester (**12**) which allowed conjugation to BSA (cGAMP-BSA). (D) an ethylenediamine-cGAMP analog conjugated to Cy5 was synthesized to be used as the fluorescently labelled cGAMP analogue in the FP assay.
