# Supplementary material for: Discovery of PF-06928215 as a high affinity inhibitor of cGAS enabled by a novel fluorescence polarization assay
Source: PLoS One. 2017 Sep 21;12(9):e0184843. doi: 10.1371/journal.pone.0184843 (PMC5608272; doi:10.1371/journal.pone.0184843)
Supplement: S5 Fig — (A) titration of mAb 80–2 in cGAMP ELISA; (B) mAb 80–2 was preincubated with cGAMP, ATP or GTP for 1 hr prior to addition to the cGAMP-BSA coated assay plates. Binding was inhibited in a concentration-dependent manner by cGAMP, but neither ATP nor GTP at mM concentrations inhibited the binding of mAb 80–2 to BSA-cGAMP. Data points are average of duplicate determinations; error bars represent standard deviation. (DOCX) [file pone.0184843.s005.docx]

**
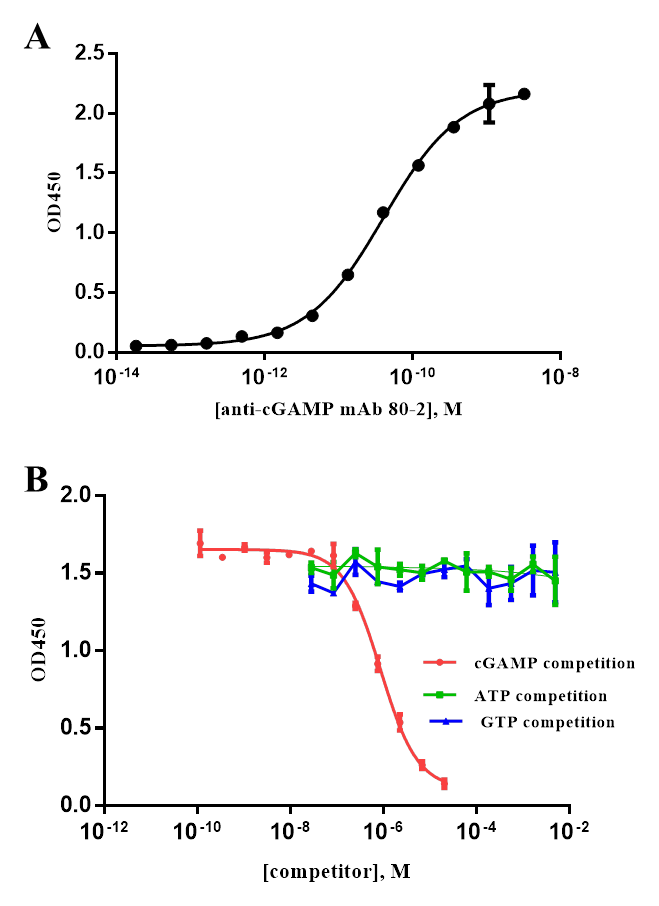
**

**S5 Figure. cGAMP mAb 80-2 characterization.** (A) titration of mAb 80-2 in cGAMP ELISA; (B) mAb 80-2 was preincubated with cGAMP, ATP or GTP for 1 hr prior to addition to the cGAMP-BSA coated assay plates. Binding was inhibited in a concentration-dependent manner by cGAMP, but neither ATP nor GTP at mM concentrations inhibited the binding of mAb 80-2 to BSA-cGAMP. Data points are average of duplicate determinations; error bars represent standard deviation.
