# Supplementary figures and images for: Discovery of PF-06928215 as a high affinity inhibitor of cGAS enabled by a novel fluorescence polarization assay
Source: PLoS One. 2017 Sep 21;12(9):e0184843. doi: 10.1371/journal.pone.0184843 (PMC5608272; doi:10.1371/journal.pone.0184843)

**
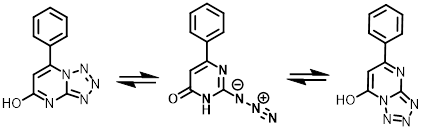
**

**S6 Figure. Compound 15 can readily isomerize via ring opening through an open azidopyrimidine**

Supplement: S6 Fig — (DOCX) [file pone.0184843.s006.docx]

**S1 Table. Crystallographic data and refinement statistics**


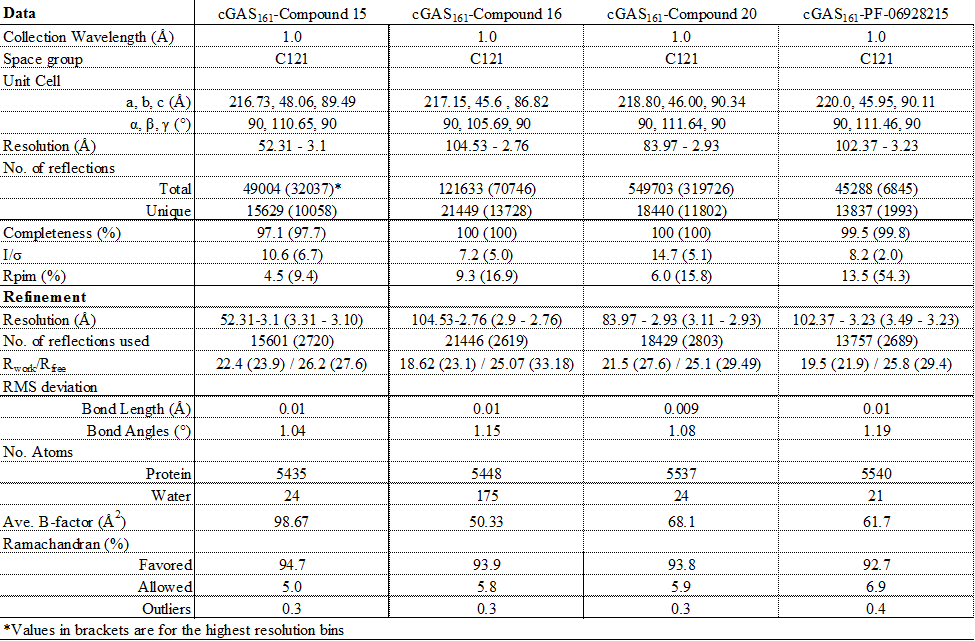

Supplement: S1 Table — (DOCX) [file pone.0184843.s008.docx]
