# Supplementary material for: Discovery of PF-06928215 as a high affinity inhibitor of cGAS enabled by a novel fluorescence polarization assay
Source: PLoS One. 2017 Sep 21;12(9):e0184843. doi: 10.1371/journal.pone.0184843 (PMC5608272; doi:10.1371/journal.pone.0184843)
Supplement: S7 Fig — THP-1 Dual cells were pretreated with various concentrations of BX-795 (red triangles), Compound 17 (yellow squres), Compound 18 (purple triangles), Compound 19 (black triangles) or PF-06928215 (blue circles) for 1 hr followed by stimulation with salmon sperm DNA for 12 hrs. Media was collected and analyzed for luciferase signal (A), and cell viability (B) was analyzed with CellTiter Glo, as described in Methods. (DOCX) [file pone.0184843.s007.docx]

**
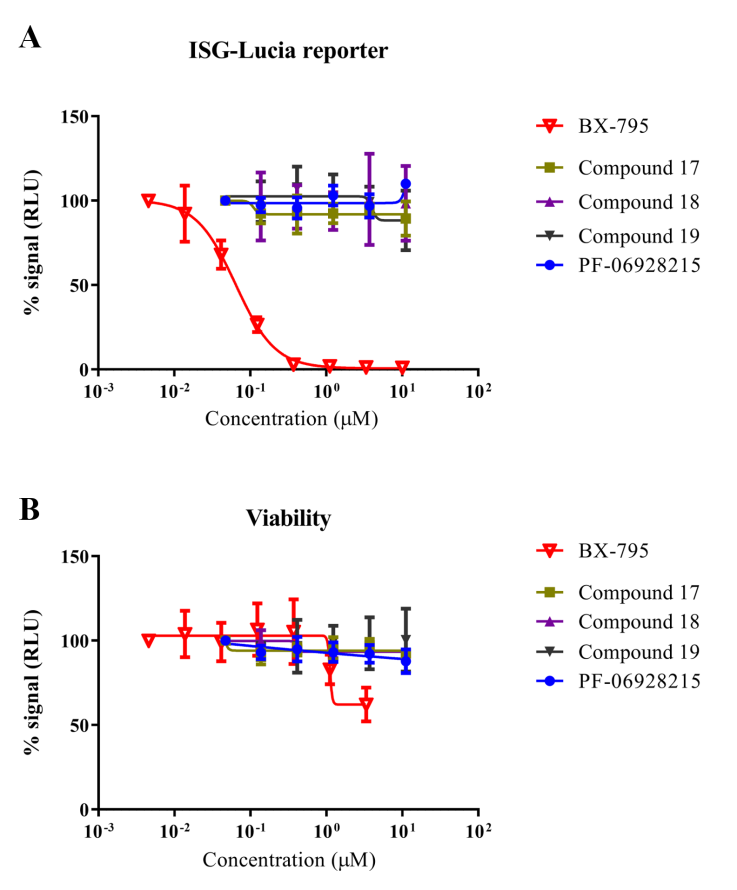
**

**S7 Figure. Effect of cGAS inhibitors on IFN β induction.** THP-1 Dual cells were pretreated with various concentrations of BX-795 (red triangles), Compound 17 (yellow squres), Compound 18 (purple triangles), Compound 19 (black triangles) or PF-06928215 (blue circles) for 1 hr followed by stimulation with salmon sperm DNA for 12 hrs. Media was collected and analyzed for luciferase signal (A), and cell viability (B) was analyzed with CellTiter Glo, as described in Methods.
